# Advances in machine and deep learning for ECG beat classification: a systematic review

**DOI:** 10.3389/fdgth.2025.1649923

**Published:** 2025-11-27

**Authors:** Allam Jaya Prakash, Abdelkader Nasreddine Belkacem, Ibrahim M. Elfadel, Herbert F. Jelinek, Mohamed Atef

**Affiliations:** 1 Electrical and Communication Engineering Department, College of Engineering, United Arab Emirates University, Abu Dhabi, United Arab Emirates; 2 Department of Computer and Network Engineering, College of IT, United Arab Emirates University, Abu Dhabi, United Arab Emirates; 3 Center for Cyber-Physical Systems and Department of Computer and Information Engineering, Khalifa University, Abu Dhabi, United Arab Emirates; 4 Department of Medical Sciences, Khalifa University of Science and Technology, Abu Dhabi, United Arab Emirates

**Keywords:** arrhythmia, classification, deep learning, electrocardiogram, feature extraction, machine learning

## Abstract

The electrocardiogram (ECG) is an important tool for exploring the structure and function of the heart due to its low cost, ease of use, efficiency, and non-invasive nature. With the rapid development of artificial intelligence (AI) in the medical field, ECG beat classification has emerged as a key area of research for performing accurate, automated, and interpretable cardiac analysis. According to the Preferred Reporting Items for Systematic Reviews and Meta-Analyses criteria, we examined a total of 106 relevant articles published between 2014 and 2024. This study investigates ECG signal analysis to identify and categorize various beats with better accuracy and efficiency, by emphasizing and applying vital pre-processing techniques for denoising the raw data. Particular attention is given to the evolution from traditional feature-engineering methods toward advanced architectures with automated feature extraction and classification, such as convolutional neural networks, recurrent neural networks, and hybrid frameworks with attention mechanisms. In addition, this review article investigates the common challenges observed in the existing studies, including data imbalance, inter-patient variability, and the absence of unified evaluation metrics, which restrict fair comparison and clinical translation. To address these gaps, future research directions are proposed, focusing on the development of standardized multi-center datasets, cross-modal fusion of physiological signals, and interpretable AI models to facilitate real-world deployment in healthcare systems. This systematic review provides a structured overview of the current state and emerging trends in ECG beat classification, offering clear insights for researchers and clinicians to guide future advancements in intelligent cardiac diagnostics.

## 1 Introduction

The electrocardiogram (ECG) signal is a crucial non-invasive tool for diagnosing and monitoring cardiac disorders (1). Its quick and accurate results make it valuable in various clinical settings (1, 2), allowing healthcare providers to assess heart rate (HR), rhythm, and conduction mechanisms (2, 3). An ECG is commonly used to screen patients with risk factors such as hypertension, diabetes, or a family history of heart disease, as minor irregularities may signify a higher risk (4). It also reveals heart size, thickness, and blood supply, helping to detect conditions such as heart failure or cardiomyopathy. In addition, an ECG is utilized during procedures or serious illnesses to monitor heart function and detect abnormal rhythms, allowing for prompt intervention (2). According to the World Health Organization (WHO), approximately 17.90 million deaths worldwide are caused by cardiovascular diseases (CVDs) each year (5). The most common CVDs (6) are arrhythmia, myocardial infarction (MI), congestive heart failure, rheumatic heart disease, cardiomyopathy, ischaemia, and heart stroke. ECGs play a vital role in diagnosing and monitoring CVDs. Therefore, a timely diagnosis and accurate ECG beat detection in cardiac patients are crucial. Identifying the morphological similarities among the many ECG beats from different classes is difficult when using the naked eye. Therefore, an automated diagnostic tool for ECG beat classification is required (7).

**Figure 1:**
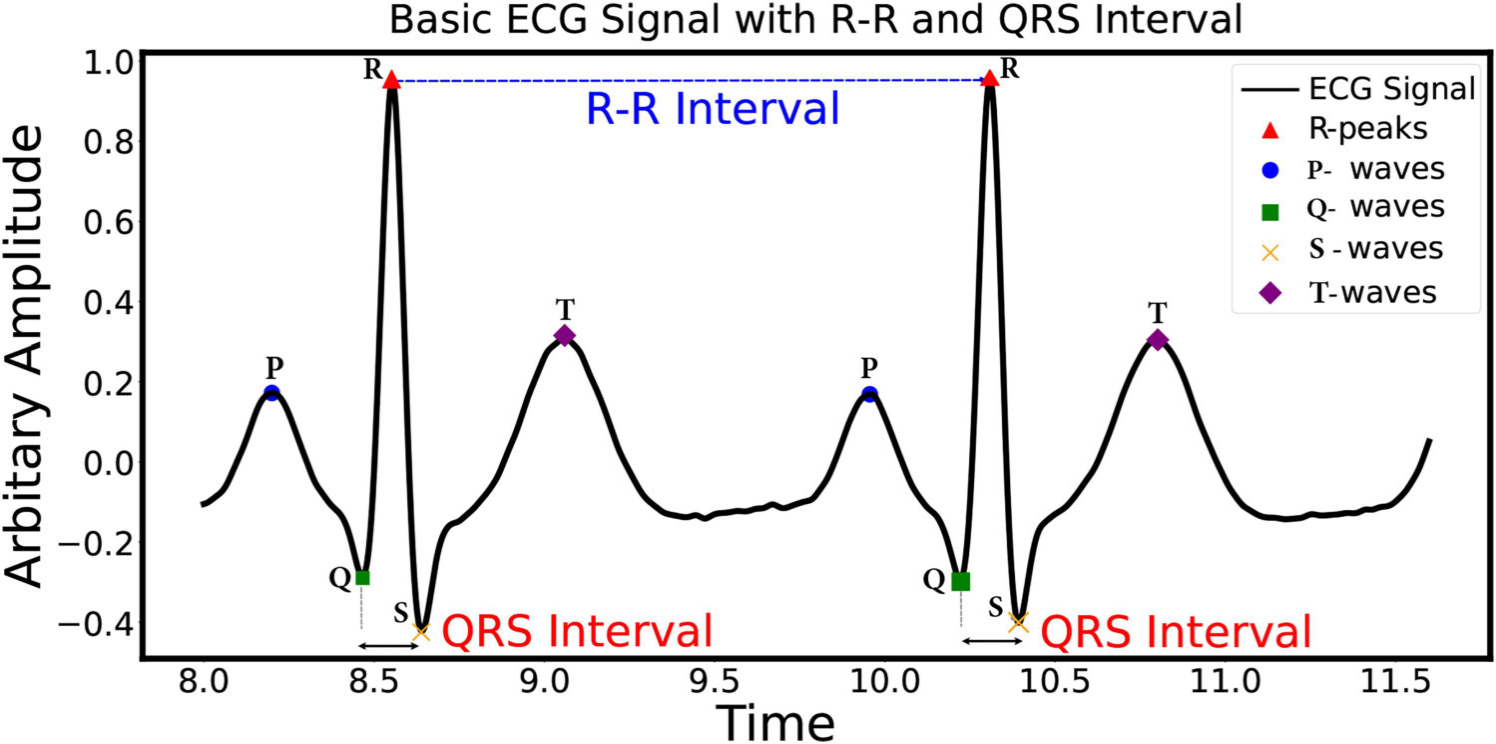
Basic ECG signal and characteristic wave representation.

Figure 1 illustrates the primary ECG signal, composed of the following characteristic waves: the P-wave, QRS complex, T-wave, and U-wave (1). These characteristic waves are crucial for identifying the state of the heart’s functioning. As per the Physionet arrhythmia database, there are 17 different types of ECG beats. These categories cover a wide range of arrhythmias, aiding in the comprehensive analysis and classification of ECG beats (8). These 17 different types of ECG beats are further sub-categorized into five classes, i.e., non-ectopic beat, supraventricular ectopic beat (S), ventricular ectopic beat (V), fusion beat (F), and unknown beat (Q), as per the American Association for the Medical Instrumentation (AAMI) (8). Among these ECG beats, S and V are clinically crucial, as these are sources of sudden heart attacks (9).

### 1.1 Automated ECG beat classification

ECG recordings are classified into two types based on the recording duration, i.e., resting and ambulatory ECG (10). A resting ECG contains only 5 to 10 min of heart function data recording, whereas an ambulatory ECG records 24–48 h of information (11). Detecting abnormal episodes from this enormous quantity of data is very difficult. Therefore, effective automated diagnosis tools are required to detect important episodes in cardiac patients. Initially, we used template-based and rule-based techniques that are usually utilized to detect the type of ECG beat (12–17). Rule-based approaches rely on predefined rules and thresholds to classify ECG beats. These rules are often based on expert knowledge or heuristics. However, these rules may not be able to handle the wide range of variations and complexities observed in real-world ECG signals. As a result, rule-based approaches may struggle to adapt to different types of beats or handle new patterns that were not considered during rule creation (18). Developing accurate and comprehensive rules for ECG beat classification can be challenging. It requires a deep understanding of ECG signal characteristics and considerable domain expertise. Designing rules that cover all possible scenarios and variations is complex and time-consuming. Rule-based approaches are typically designed to classify beats based on specific features or patterns (19). They may struggle to generalize well to new or unseen data that do not conform to the predefined rules. The rule-based classifier can produce incorrect or inconsistent results if the ECG data deviates from the expected patterns. A set of templates representing different types of beats is required in template-based approaches (13). Choosing appropriate templates that accurately represent the various beat morphologies in ECG signals can be challenging. There is a need to consider inter-subject and intra-subject variability and variations due to different conditions and diseases (12). The classification in template-based approaches relies on comparing the input ECG beat with a set of templates to find the best match. However, template matching can be sensitive to noise, baseline wander (BW), and other artifacts present in the signal (20). These issues can affect the accuracy of the match and lead to misclassification. Template-based approaches often struggle with scalability when dealing with large datasets or real-time applications (17). Comparing each beat with a set of templates can be computationally expensive, especially if the number of templates is high. The time complexity increases as the number of beats and templates grows, making it impractical for large-scale applications (21).

### 1.2 Machine and deep learning for ECG beat classification

**Figure 2:**
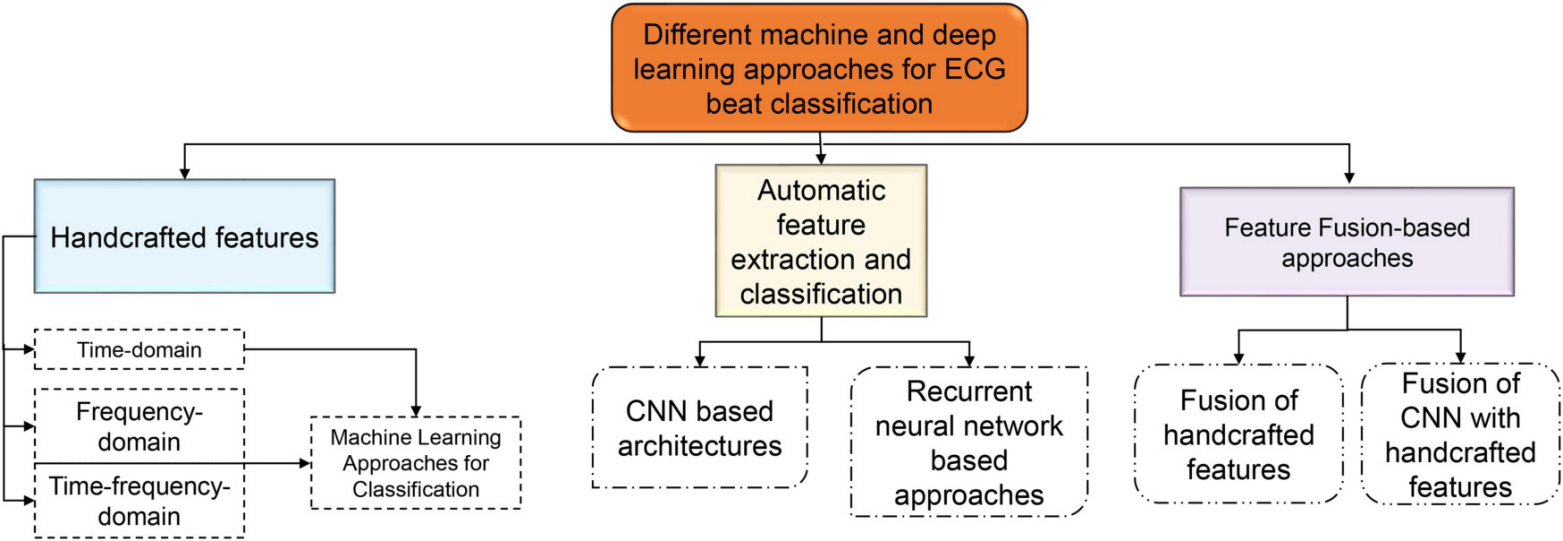
Hierarchical representation of the state-of-the-art ECG beat classification techniques.

Machine learning (ML) algorithms are becoming popular for effective classification of ECG beats (18, 19, 22–28). Hierarchical representations of the latest ECG beat classification techniques based on machine and deep learning (DL) techniques have been reported in the literature, as shown in Figure 2. Handcrafted features are required to detect the type of ECG beat when using machine learning techniques (22–24). Most handcrafted features are extracted based on time, frequency, and time-frequency domains (29, 30). Time-domain features alone are not sufficient for effective ECG beat classification; together with this, the frequency-domain features increase beat detection performance (11, 29). In addition, features based on time and frequency are more effective than individual features in the time and frequency domain (31). Handcrafted approaches rely on manual feature engineering, where domain knowledge is used to design and extract features from ECG signals. This process can be time-consuming, involving the design of algorithms and signal processing techniques to extract meaningful features (5). Handcrafted approaches require selecting relevant features that capture the discriminative information from the ECG signals. Identifying the most informative and robust features is non-trivial and often requires domain expertise (32). Choosing inappropriate features or excluding important ones can lead to suboptimal classifier performance. Handcrafted feature sets may not generalize well to new or unseen data that significantly differ from the training data. ECG signals vary considerably due to age, sex, underlying conditions, and noise (33). The classification accuracy may be compromised if the handcrafted features do not represent the new data. ECG beat classification involves capturing complex relationships and patterns within signals. Handcrafted approaches may struggle to capture these intricate relationships, as they typically rely on pre-defined algorithms and feature engineering techniques. These problems have motivated researchers to develop automatic feature extraction approaches.

Deep learning models can automatically learn relevant features directly from the raw ECG signals, eliminating the need for manual feature engineering. This feature learning process enables the model to capture intricate patterns and complex relationships that may be difficult to capture using handcrafted features (18, 19, 25–28). Deep learning models can learn hierarchical representations of ECG signals, allowing them to extract meaningful features at different levels of abstraction. The different deep learning architectures and their advantages in the extraction of meaningful features are presented in Table 1. Deep learning models are known for generalizing unseen data well. They can learn from large amounts of labeled ECG data and capture the underlying patterns that are characteristic of different beat types. Deep learning models can handle large-scale ECG datasets efficiently (20). Once trained, the models can process ECG beats quickly, making them suitable for real-time applications. In addition, deep learning models can be deployed on parallel computing architectures, such as graphical processing units (GPUs), to improve computational performance, enabling rapid and scalable ECG beat classification (9, 18). Deep learning models can be updated and fine-tuned with new data to improve performance and adapt to changes in ECG signals (25). This ability for continual learning allows the model to incorporate new knowledge and adjust its classification capabilities as new data become available. It enables the model to stay up-to-date with the emerging beat types or changes in the data distribution (28). Deep learning models can adapt and generalize well to new beat types and variations not encountered during training. The models can discover and classify novel patterns and variations by learning from diverse examples. This adaptability makes deep learning models suitable for dynamic environments where ECG data may evolve.

**Table 1:** Hierarchy of different deep learning architectures for ECG beat classification.

Architecture	Layers	Advantages	Disadvantages	Remarks
Convolutional neural network (CNN)	Convolutional, pooling, fully connected layers	Excellent for spatial feature extraction; robust against noise with adequate preprocessing	Requires large labeled datasets; performance degrades on noisy data	Suitable for clean, structured ECG datasets
Recurrent neural network (RNN)	Recurrent layers (e.g., GRU, LSTM)	Captures sequential dependencies; handles temporal features effectively	Prone to vanishing gradient problems; slow training process	Useful for time-series analysis but requires gradient management
Long short-term memory (LSTM)	Input, forget, output gates	Solves vanishing gradient problem; excellent for long-term dependencies	Computationally expensive; sensitive to hyperparameter tuning	Ideal for sequential tasks such as ECG signal interpretation
Bi-directional LSTM (BiLSTM)	Forward and backward LSTM layers	Captures past and future temporal features	High computational overhead; challenging for large datasets	Performs well in tasks requiring bidirectional dependencies
Hybrid CNN-LSTM	Convolutional layers + LSTM layers	Combines spatial and temporal feature learning	High computational cost; pre-processing (e.g., QRS detection) required	Effective for complex ECG signal classification
Dense convolutional network (DenseNet)	Dense convolutional blocks, transition layers	Efficient feature reuse; excellent for structured data	High memory requirement due to dense connections	Ideal for datasets with detailed temporal-spatial patterns
Residual networks (ResNet)	Residual blocks with skip connections	Solves degradation problem; enables deeper networks	Requires careful tuning for small datasets	Useful for capturing subtle variations in ECG signals
Autoencoders	Encoder–decoder architecture	Learns unsupervised representations; useful for anomaly detection	Limited interpretability; prone to over-fitting	Ideal for anomaly detection in ECG signals
Attention mechanism	Attention layers on top of RNN or CNN	Focuses on critical parts of the ECG signal	Computationally expensive; complex to train	Effective for applications requiring interpretability
Transformer networks	Multi-head attention, positional encoding	Handles long-range dependencies; scalable for large datasets	Requires large datasets and high computational resources	Promising for real-time, large-scale ECG classification

The remainder of this article is organized as follows. Section 2 discusses the main objectives and methodology of the proposed study. Section 3 refers to the publicly available databases for experimentation on ECG beat classification. The background and significance of ECG beat classification are discussed in Section 4, with pre-processing, feature extraction techniques, and ECG beat classification algorithms, along with their performance, elaborated on in Sections 4.1–4.3. A general discussion of machine learning and deep learning for the detection of ECG beats is presented in Section 5. Finally, limitations, future directions, recommendations from the state-of-the-art review, and conclusions are presented in Sections 6, 8, and 9.

## 2 Systematic review protocol

The primary focus of this research was to thoroughly explore the extensive range of machine learning and deep learning methodologies employed in the context of ECG beat classification. Arrhythmia is a condition characterized by irregular heartbeats, either too fast or too slow, that disrupt the normal functioning of the heart (2). The heart has a natural pacemaker that sets the rhythm of the heartbeats. However, any disturbances in electrical impulses can result in arrhythmia. There are many cardiac arrhythmias, each with unique characteristics and potential complications. Early detection and treatment of arrhythmias can help prevent serious complications such as stroke or sudden cardiac arrest. Significant cardiac arrhythmias include (i) atrial fibrillation (AF), (ii) ventricular tachycardia (VT), (iii) sinus bradycardia (SB), (iv) atrial flutter (AFT), (v) ventricular fibrillation (VF), and (vi) supraventricular tachycardia (SVT) (34). An irregularity in the upper chambers of the heart causes AF, which in turn causes blood clots, stroke, and heart failure (35). A fast heart rate that starts in the ventricles (the lower chambers of the heart) will produce VT beats. It can cause dizziness, chest pain, and fainting. If left untreated, it can also lead to sudden cardiac arrest. SB is a slow heart rate that originates in the sinoatrial node (SA). It can cause fatigue, dizziness, and fainting. AF is an irregular heart rhythm caused by electrical activity in the atrial chambers of the heart. The atrial rate is generally between 250 and 350 beats per minute and is quick and regular. Since the atrioventricular (AV) node slows down electrical impulses, the ventricles (the lower chambers of the heart) can also beat rapidly (34).

The study investigates ECG signal analysis to identify and categorize various ECG beats with better accuracy and efficiency. The investigation emphasizes vital pre-processing techniques for denoising the raw ECG data to achieve this objective. The removal of unwanted noise ensures that subsequent classification algorithms can work with high-quality input, ultimately leading to more robust and reliable results. These pre-processing steps are essential in enhancing the performance of the classification models reviewed in this study (21, 29, 36, 37). Furthermore, the review study in this work explores various feature extraction and selection techniques (11, 35, 36). These methods are fundamental in transforming the raw ECG signals into a set of discernible and informative features that the classification algorithms can effectively utilize. By studying and comparing the various feature extraction methods available in the literature, this study aimed to identify the most relevant and influential features in the classification of ECG beats, with the aim of optimizing the precision and efficiency of the classification process (19, 20, 33). This study also surveys and discusses the existing literature, critically assessing the performance of the different machine and deep learning techniques employed in ECG beat classification. By evaluating the strengths and weaknesses of these methods, the research aims to provide valuable information on the most promising approaches and their potential applications in real-world scenarios, such as arrhythmia detection and cardiac health monitoring (2). Moreover, this investigation seeks to contribute to the broader field of biomedical signal processing by presenting a comprehensive overview of the state-of-the-art techniques for ECG beat classification (18, 19, 25–27, 38–40). By consolidating and presenting this knowledge, researchers and practitioners can better understand the most effective methodologies available and further advance the field’s capabilities.

### 2.1 Search strategy: inclusion and exclusion criteria

**Figure 3:**
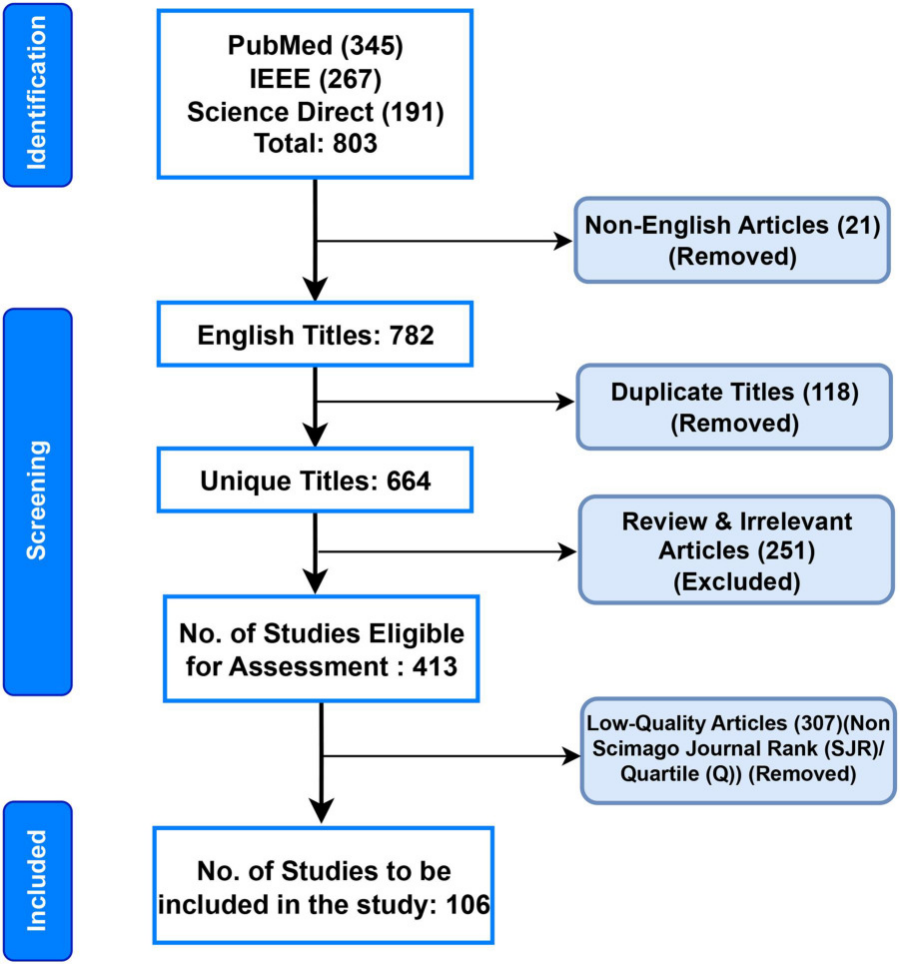
Flow diagram for the systematic review of ECG beat classification, following the PRISMA guidelines.

**Figure 4:**
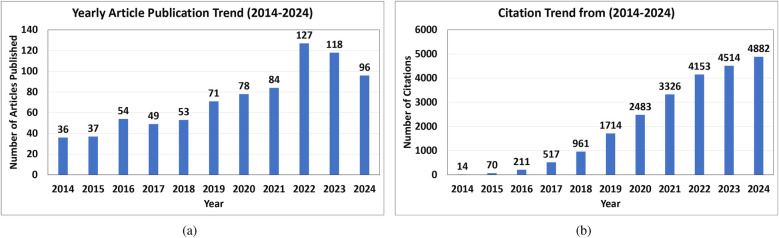
**(a)** Article and **(b)** citation trends for ECG beat classification from 2014 to 2024.

We used the Preferred Reporting Items for Systematic Reviews and Meta-Analyses (PRISMA) criteria to find studies pertinent to the classification of ECG beats (41). Articles published up until April 2024 utilizing the terms “Electrocardiogram,” “ECG beat Classification,” “Artificial Intelligence,” “Machine Learning,” and “Deep Learning” in their respective Boolean combinations were searched for in the PubMed, Institute of Electrical and Electronics Engineers Association (IEEE), and Science Direct databases. The authors excluded non-English publications, duplicate titles, irrelevant works, review articles, pilot studies, non-accessible articles, and articles published before 2014 (42). Thus, this study consisted of 106 articles that focused on AI-based ECG beat classification. Figure 3 shows the detailed search method, which was compliant with the PRISMA criteria (42). In Figure 4, it can be observed that the yearly article publication trend (2014–2024) and yearly citation trend (2014–2024) for ECG beat classification indicate a strong and growing research interest in this domain. The publication trend shows a steady increase in the number of articles, which peaked in recent years (2022–2024), highlighting the expanding focus on deep learning, signal processing, and patient-specific classification approaches. This growth suggests an increasing number of researchers contributing to advancements in ECG analysis. A peak in citations often follows high publication periods, indicating that time is required for newer methods to be widely cited. These trends emphasize the need for continued innovation and the adoption of novel approaches to sustain research in ECG beat classification.

To ensure the inclusion of high-quality and peer-reviewed research, the study employed a quality-filtering criterion based on the Scimago Journal Rank (SJR) and Journal Citation Reports (JCR) quartile (Q) indexing systems. In this review, only articles published in journals indexed in SJR or those with JCR quartile rankings (Q1–Q4) were considered eligible for inclusion. Studies published in journals not indexed in either SJR or JCR, or lacking identifiable quality metrics, were classified as “Non-SJR/Q” and consequently excluded. The SJR metric reflects the scientific influence of scholarly journals by accounting for both the number of citations received and the prestige of the citing journals, while the JCR-Q system ranks journals from Q1 (highest impact) to Q4 (lowest) based on citation distributions. This quality screening ensured that the included literature represented peer-reviewed, credible, and widely recognized sources within the scientific community. Furthermore, conference papers, pilot studies, non-English articles, and inaccessible manuscripts were excluded to maintain methodological rigor and focus on reproducible, peer-reviewed work.

**Figure 5:**
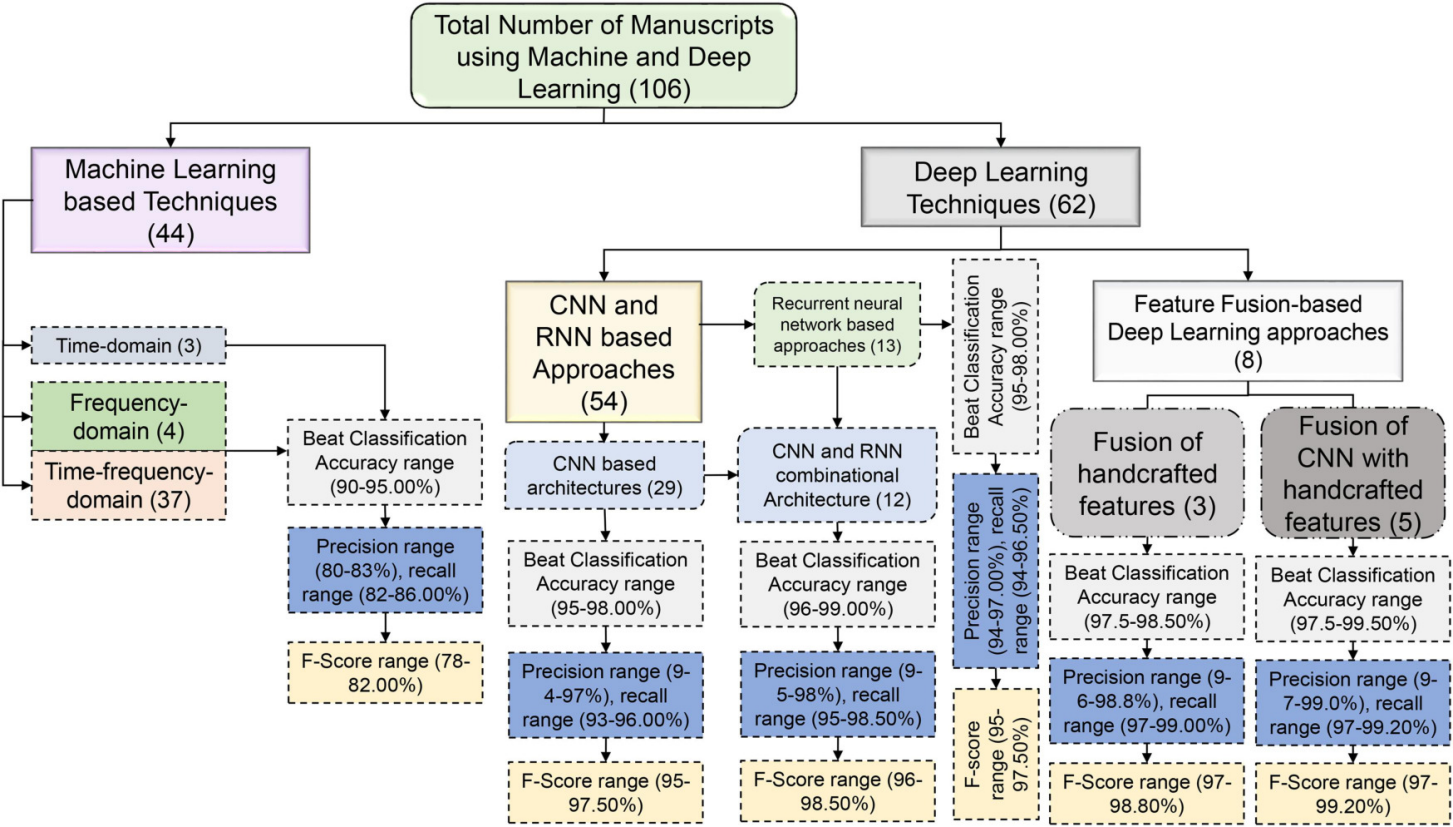
Manuscript details and performance ranges of different machine and deep learning techniques.

Figure 5 summarizes the most commonly reported performance indicators, namely, accuracy, precision, recall, and F-Score. It is acknowledged that these metrics can be misleading under severe class imbalance. Accuracy, in particular, tends to overestimate performance when normal beats dominate the dataset. Although several reviewed studies reported more robust and threshold-independent metrics, such as the Matthews correlation coefficient (MCC) and area under the precision-recall curve (AUPRC), these measures were not consistently available across all publications, preventing their inclusion in the aggregated summary plots. To maintain comparability across the 106 reviewed works, we therefore only visualized the universally reported metrics. Nevertheless, we recognize that MCC and AUPRC provide a more balanced and informative assessment of classifier performance, especially for minority arrhythmic classes such as supraventricular ectopic beat (SVEBs) and ventricular ectopic beat (VEB). This limitation highlights the need for future ECG classification studies to adopt standardized, imbalance-aware metrics to enable more equitable and clinically meaningful performance comparisons. Initially, machine learning methods relied heavily on manual feature extraction, where domain experts would identify relevant characteristics of ECG signals, such as QRS complexes, heart rate variability, and waveform shapes (29, 33, 43–46). These handcrafted features were then fed into classifiers, such as support vector machine (SVM), random forest (RF), and k-nearest neighbors (kNN), which, despite their effectiveness, were often limited by the quality and completeness of the extracted features. With the advent of deep learning, the process became more automated and data-driven, allowing models such as CNNs and RNNs to learn directly from raw ECG data (25–27). These networks captured complex temporal dependencies and subtle morphological variations without explicit feature engineering, resulting in more robust and accurate ECG beat classification systems. This shift improved classification performance and opened new possibilities for real-time and patient-specific ECG analysis.

## 3 Data sources

In the literature, the majority of the studies have used three different ECG databases to verify how effectively ECG beat classification methods perform, namely, the Massachusetts Institute of Technology—Beth Israel Hospital (MIT-BIH) arrhythmia (8), St. Petersburg Institute of Cardiological Technics (INCART) (8), and MIT-BIH supraventricular arrhythmia (3) databases. A summary of these three databases is shown in Table 2. Table 3 presents a detailed breakdown of the manuscripts that utilized various ECG beat classification databases. It further highlights the use of the three prominent databases—MIT-BIH Arrhythmia, INCART, and MIT-BIH supraventricular—across three major publishers: Elsevier, IEEE, and Springer. In addition, it includes lesser-known databases such as the American Heart Association (AHA) and MIT-BIH Long-Term ECG, which are used for specialized research in arrhythmia detection. This table helps to identify trends in database usage for ECG classification in the scientific community.

**Table 2:** Details of the databases utilized for ECG beat classification.

S.no	Name of the database	Number of subjects	Sampling frequency (in Hz)	Resolution	Total number of ECG segments	No. of classes
1	MIT-BIH Arrhythmia (8)	47	360	11-bits	109,000	17
2	St Petersburg INCART (8, 47)	25	257	12-bits	175,840	15
3	MIT-BIH Supraventricular Arrhythmia (3, 47)	14	128	11-bits	184,508	9

**Table 3:** Estimated number of manuscripts using various ECG beat classification databases from different publishers.

Database	Elsevier manuscripts	IEEE manuscripts	Springer manuscripts
MIT-BIH Arrhythmia Database(8)	22	18	19
INCART (8, 47)	5	7	7
MIT-BIH Supraventricular Database(47)	11	9	8
PTB diagnostic ECG (47)	2	3	9
European ST-T (47, 48)	4	3	5
LTAF (long-term AF) (47, 49)	5	6	3
Atrial Fibrillation Database (AFDB) (47, 50)	6	8	4
CPSC (China Physiological Signal Challenge) (51)	4	5	2
CINC (Computers in Cardiology Challenge) (47, 52)	3	4	7
Others	6	9	9

*MIT-BIH Arrhythmia Database (8):* The MIT-BIH arrhythmia database includes both typical and abnormal cardiac rhythms. This database is widely considered the gold standard in heartbeat detection and classification. There are 48 ambulatory ECG recordings, each lasting 30 min and collected from 47 subjects (8). The first 23 recordings are connected to standard clinical recordings, whereas the rest feature potentially fatal cardiac arrhythmias. Each ECG signal is collected at 360 Hz in these data (8). There are two information streams in every recording, with the first channel’s signal (MLII) being of higher quality than the second (V5). There are around 1,09,000 heartbeats in the collection, annotated with 16 distinct labels. Four records (102, 104, 107, and 217) are of inadequate quality out of 48 ECG records (8). As a result, the classification efficiency is calculated without including these files. The ECG signals in the MIT-BIH Arrhythmia Database were recorded from different patients, and even for the same patient, the signal characteristics can vary over time. This can make it challenging to develop a generalized classification system that can perform well on new patients (8).

*St Petersburg INCART Arrhythmia Database (47):* One popular dataset for training and testing ECG classification algorithms is the 12-lead arrhythmia dataset from the St. Petersburg INCART database. Arrhythmias, ischaemic heart disease, myocardial infarction, and other cardiac diseases are represented in the seventy-five 15 min ECG recordings from 25 patients (53). Two cardiologists have manually annotated each recording with beat annotations in the database. The reference annotation files have more than 175,000 beat annotations, which are useful for testing and developing ECG classification systems. Although the patient group is diverse in age, gender, and disease, it may not represent all patient populations (53).

*MIT-BIH Supraventricular Arrhythmia Database (3):* This dataset includes 78 two-lead ECG recordings, each lasting for 30 min. The recordings were collected from 14 patients, 11 men and three women, with different types of supraventricular arrhythmias (3). These included normal, ventricular, fusion, and unknown beats. The ECG signals were sampled at 128 Hz and digitized with 11-bit resolution. Physicians and researchers have extensively utilized this database to develop and validate algorithms to detect and classify various arrhythmias (3). It has been used in several studies to compare the performance of different algorithms, including machine learning and deep learning algorithms (28).

### 3.1 Data imbalance issues in the above databases

Data imbalance is a significant challenge in ECG beat classification, particularly when using the MIT-BIH Arrhythmia (8), St. Petersburg INCART (8, 47), and MIT-BIH Supraventricular Arrhythmia (47) databases. These databases contain varied distributions of ECG beat types, often resulting in an overrepresentation of normal or common beats and an underrepresentation of rare arrhythmias, which may degrade the model performance and reduce generalizability. In the MIT-BIH Arrhythmia database, N beats vastly outnumber abnormal arrhythmias such as VEB or SVEB (8). As a result, classifiers trained on this database often achieve high accuracy by predominantly predicting the majority class (N beats), while failing to correctly identify rarer arrhythmias. This class imbalance can lead to a high false-negative rate for life-threatening conditions such as ventricular tachycardia, as the classifier may not learn sufficient patterns for these rare events. Techniques, such as the oversampling of minority classes and undersampling of the majority class, and applying synthetic data generation methods, such as the synthetic minority oversampling technique (SMOTE), can help mitigate this imbalance (54). In addition, cost-sensitive learning, where higher penalties are assigned to misclassifying rare classes, can also improve classification performance.

The INCART database, which includes recordings from 25 subjects with different types of arrhythmias, also suffers from data imbalance (8, 47). The majority of the beats in this dataset are NSR, while pathological beats such as ischaemic events are underrepresented. Similar to the MIT-BIH Arrhythmia database, this imbalance can bias classifiers towards predicting normal beats. Addressing this issue is crucial for the real-world application of ECG beat classifiers, as it ensures the model can detect critical events such as ischaemia. To address this imbalance, data augmentation techniques, such as adding noise or time-shifting the ECG signals of rare arrhythmias, can be used, as well as balancing the dataset through stratified sampling (55). The MIT-BIH Supraventricular Arrhythmia database also presents a unique challenge due to its smaller dataset size and the imbalance between normal and supraventricular arrhythmias (47). Given the relatively low number of supraventricular beats compared to normal beats, classifiers can become biased towards predicting normal rhythms, further complicating the detection of less frequent but clinically important supraventricular ectopic beats.

Bias in machine learning refers to systematic errors that cause a model to make consistently incorrect predictions, often favoring certain patterns while neglecting others (56). Bias can arise due to several factors, such as imbalanced training data, poor feature selection, or overly simplistic model assumptions that fail to capture the true complexity of the data (19). Bias in ECG beat classification is a critical issue, often stemming from imbalanced datasets where certain heartbeat types are overrepresented while others are underrepresented. This imbalance can lead to classifiers favoring the majority class, resulting in poor sensitivity for minority classes, such as supraventricular and fusion beats (57). One study demonstrated that standard classification models trained on imbalanced datasets tend to overfit the majority class while failing to recognize less frequent arrhythmias effectively (58). This issue is exacerbated by linear dependencies in ECG data, which further skew the model’s learning process and introduce classification bias (57). Moreover, the impact of inter-patient variability, where models perform well on seen patients but fail to generalize to unseen cases, further contributes to biased ECG classification (59). A detailed overview of certain issues due to bias is presented in Table 4.

**Table 4:** Bias issues and solutions in ECG classification.

Literature	Issue	Impact	Solution
(57)	Imbalanced datasets	Certain heartbeat types are overrepresented, leading to classifiers favoring majority classes and poor sensitivity for minority beats	Dynamic minority-biased batch weighting loss function to prioritize underrepresented classes
(58)	Overfitting to majority class	Standard models fail to recognize less frequent arrhythmias effectively, resulting in classification errors	Feature fusion neural networks to extract diverse feature sets and improve classification fairness
(57)	Linear dependencies in data	Skews the model’s learning process and increases classification bias, making it harder to detect minority classes	Differential beat accuracy (DBA) metric to optimize classifier learning based on data distribution
(59)	Inter-patient variability	Models perform well on seen patients but fail to generalize to new, unseen cases, reducing real-world effectiveness	Ensemble-based classification techniques to improve generalization by leveraging diverse model outputs
(98)	Cost-sensitive misclassification	Traditional classifiers may misclassify minority beats due to non-optimized decision boundaries, reducing clinical reliability	Cost-sensitive classifiers that adjust decision thresholds based on the misclassification cost of minority classes
(175)	Feature selection bias	Feature selection methods can introduce bias if not carefully designed, leading to suboptimal classification performance	Hybrid deep learning models combining rule-based and data-driven feature selection to minimize bias
(60)	Limited training data	ECG classification models struggle with limited labeled data, resulting in poor generalization to new datasets	Data augmentation techniques such as synthetic ECG generation to enhance training dataset diversity

To address bias in ECG beat classification, several state-of-the-art methods have been proposed (19, 58–60). One promising approach is the use of a dynamic minority-biased batch weighting loss function, which enhances the learning process for minority classes while maintaining the model’s ability to classify the majority classes accurately (59). In addition, feature fusion neural networks, which integrate multiple ECG representations, have been shown to improve classification fairness by extracting diverse feature sets that reduce bias (59). Another strategy involves differential beat accuracy (DBA), a metric that optimizes classifier performance by adjusting the learning process based on the statistical distribution of different beat types, ensuring a more balanced classification (58). Ensemble-based techniques, such as multiple-classifier architectures, have also been effective in reducing bias by leveraging diverse model outputs to correct misclassifications (60). Collectively, these approaches contribute to improving the fairness and generalization of ECG beat classification models, making them more suitable for real-world clinical applications. In addition to traditional methods for addressing imbalance, transfer learning can be a beneficial approach in this case (55). A model pre-trained on a larger, more balanced ECG dataset can be fine-tuned using the smaller MIT-BIH Supraventricular database, allowing the model to better generalize across beat types. In all these cases, proper evaluation metrics should be used to assess the classifier’s performance under imbalance conditions. Accuracy alone may not be a reliable metric, as it can be misleading in imbalanced datasets. Instead, metrics such as the F1-score, sensitivity, specificity, and AUPRC provide a more comprehensive understanding of model performance on both the majority and minority classes (61). By carefully considering these strategies and metrics, researchers can better handle the class imbalance problem in ECG beat classification, leading to more robust and clinically useful models.

## 4 Overview of included studies

**Figure 6:**
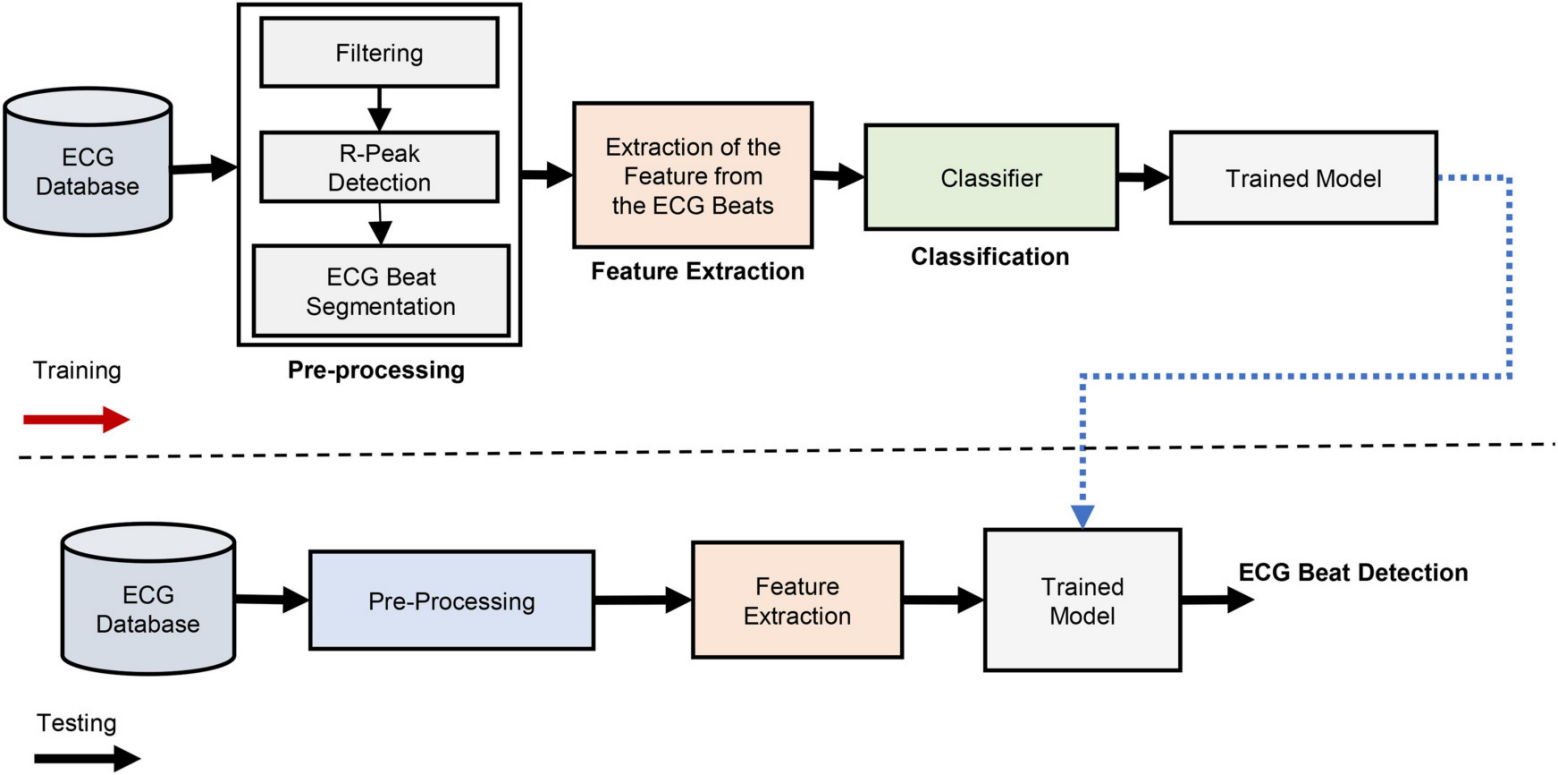
Generalized block diagram of ECG beat classification.

The ECG beat classification method is depicted as a block diagram in Figure 6. The three primary stages of ECG beat classification systems are pre-processing, feature extraction, and classification. In automated machine learning (AutoML) algorithms (11, 36, 62), feature extraction and classification are often treated as separate stages. This means the algorithm typically involves a dedicated process for extracting relevant features from the raw data, followed by a classification module that uses these features to make predictions. In contrast, deep learning algorithms often integrate feature extraction and classification into a single module. This is due to the hierarchical structure of neural networks, particularly in CNNs and other deep learning architectures, where the network learns to extract features and classify data in an end-to-end manner automatically (18, 19, 25–28). This integration is one of the key strengths of deep learning, as it eliminates the need for manual feature engineering and allows the model to optimize the feature extraction process as part of the overall learning task.

### 4.1 Pre-processing

**Figure 7:**
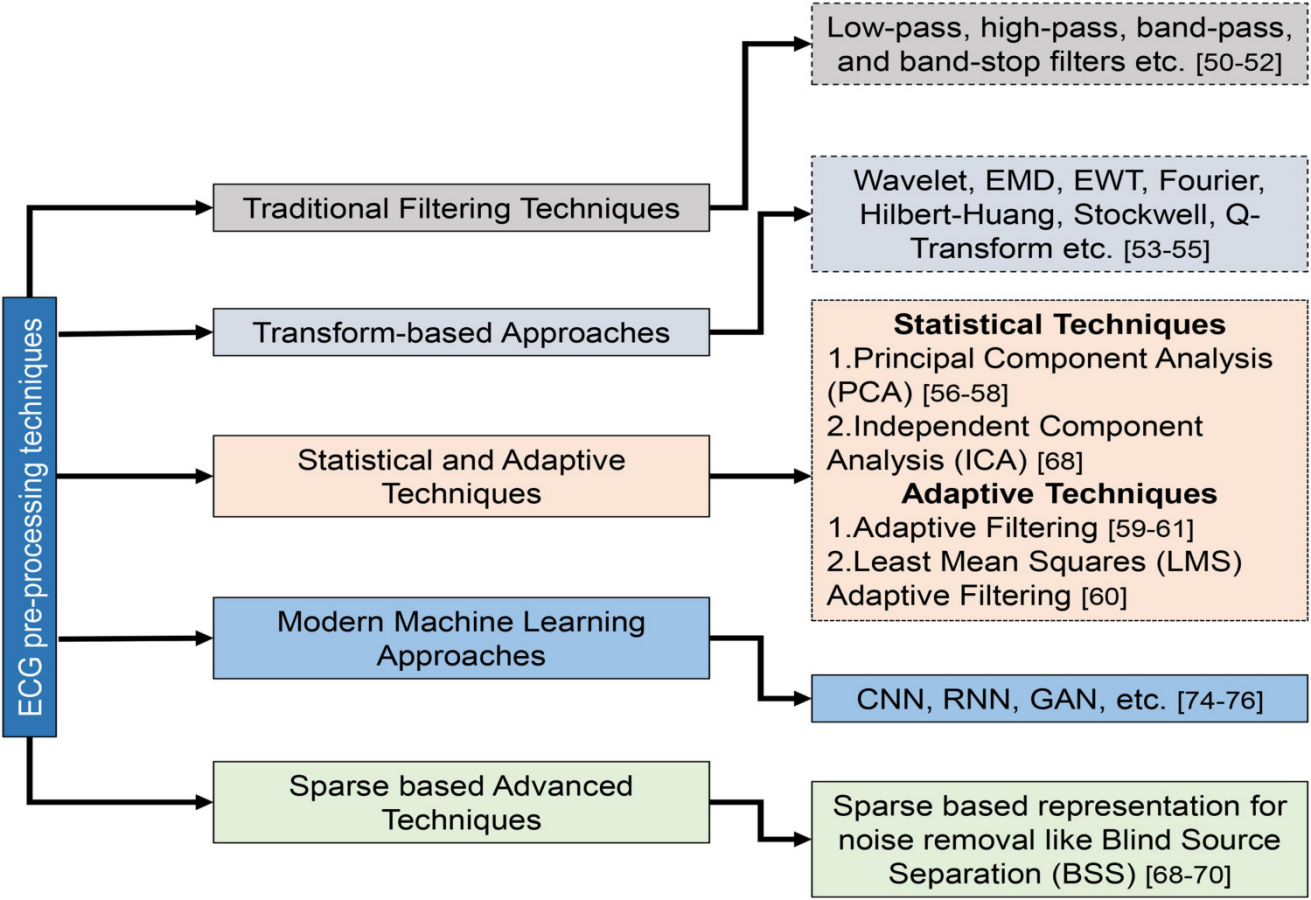
Overall classification of ECG signal pre-processing techniques.

Pre-processing is the initial and critical stage in ECG beat classification systems, aiming to enhance the quality of the raw ECG signals (Table 5). This stage is essential to mitigate the impact of noise and artifacts that can obscure the true physiological information in the ECG data. Pre-processing technique details and their categorization are illustrated in Figure 7. Common sources of noise include baseline wander, powerline interference (PLI), and muscle artifacts (MAs) (36). Techniques such as bandpass filtering, wavelet transforms, and normalization are employed to remove or reduce these interferences. In addition, this stage may involve the segmentation of the ECG signal into individual beats, setting the foundation for accurate feature extraction and classification in subsequent phases. First, the raw ECG signal is filtered to eliminate unwanted noise and artifacts in the preprocessing stage (35). The next step is to separate the signal into individual heartbeats, often identified by the R-peaks in the ECG. In the feature extraction phase, data from each heartbeat are parsed for various features based on its time domain, frequency domain, and morphology. Heartbeat rhythms and shapes are characterized using these properties, which can be utilized to identify certain arrhythmias. Classification involves applying machine learning methods to the retrieved attributes of individual heartbeats (90). The basic techniques for pre-processing ECG signals can be summarized as follows: classical filtering techniques, transform-based techniques, statistical and adaptive techniques, modern machine learning approaches, and advanced techniques.

**Table 5:** ECG signal pre-processing methods: summary, advantages, and disadvantages.

Method	Summary	Advantages	Disadvantages
Bandpass filtering (63–65)	Utilizes high-pass (HP) and low-pass filters (LPFs) to remove frequencies outside the heart’s signal range (e.g., 0.5–150 Hz). Commonly implemented using Butterworth or Chebyshev filters.	Effectively removes baseline wander and high-frequency noise (e.g., muscle artifacts).	May distort the original ECG signal, especially the QRS complex.
Wavelet transform (66–68)	Decomposes the ECG signal into different frequency components using wavelet functions such as Daubechies or Symlets.	Can localize noise in both time and frequency domains, making it ideal for isolating noise such as baseline wander and powerline interference.	Computationally intensive; selecting the appropriate wavelet function and level of decomposition can be challenging.
Principal component analysis (PCA) (69–71)	Reduces dimensionality by extracting the principal components, which retain the most significant features of the ECG signal, while discarding noise components.	Efficient in noise reduction and works well for multi-channel ECG data	Can lead to loss of important information if principal components are not correctly identified; assumes linearity
Adaptive filtering (72–74)	Uses a reference noise signal (e.g., from a secondary channel) and adapts the filter coefficients to remove the correlated noise. Common algorithms include LMS (least mean squares).	Can dynamically adjust to varying noise conditions; effective in reducing powerline interference and motion artifacts.	Requires a reference signal; may not perform well if noise is highly non-stationary.
Empirical mode decomposition (EMD) (75–77)	Breaks down the ECG signal into intrinsic mode functions (IMFs) to separate noise from the underlying signal.	Adaptive method that works well with non-linear and non-stationary signals, such as ECG data.	Can introduce mode mixing, where noise and signal are not clearly separated; sensitive to noise in the decomposition process.
Notch filtering (56, 78, 79)	A narrow-band filter is used to eliminate specific frequencies, typically used to remove powerline interference (e.g., 50/60 Hz).	Highly effective in removing powerline interference without affecting the rest of the signal.	Can distort the ECG signal around the notch frequency; it does not address other noise sources.
Sparse representation (80–82)	Represents the ECG signal as a sparse linear combination of basis functions. Noise is filtered out by removing components that do not contribute significantly to the sparse representation.	Effective in removing various types of noise while retaining signal morphology; adaptable to low-SNR ECG data.	Requires careful selection of the dictionary; computationally expensive for large datasets.
Non-local means (NLM) filtering (76, 83, 84)	Averages similar ECG signal patches to reduce random noise. The similarity between patches is determined based on their intensity and spatial distance.	Highly effective in reducing random noise while preserving the sharp features of ECG signals.	Computationally intensive, especially for large datasets, can blur features if parameter selection is improper.
Deep learning-based denoising (85–87)	Uses deep learning models such as autoencoders, CNNs, or RNNs to learn noise patterns and denoise ECG signals in a supervised or unsupervised manner.	Capable of removing complex noise patterns while preserving ECG features; adaptable to real-time applications.	Requires a large dataset for training; computationally intensive and can overfit if not properly regularized.
Total variation denoising (TVD) (88)	Reduces noise by minimizing total variation in the signal, preserving edges (e.g., sharp ECG features).	Preserves sharp transitions such as R-peaks, effectively removing low-level noise.	May lead to over-smoothing if the regularization parameter is not properly chosen.
Empirical wavelet transform (EWT) (89)	A variant of the wavelet transform that adapts the filter bank to the specific frequency bands of the input signal.	Automatically adapts to the signal’s characteristics, providing better decomposition for non-stationary signals like ECG.	Still computationally expensive and requires careful parameter tuning.
Generative adversarial networks (GAN)-based denoising (85, 86)	Employs GANs where the generator learns to denoise ECG signals while the discriminator ensures that the denoised signal is close to the true ECG.	Can learn complex noise patterns and adapt to various datasets; highly flexible.	Training GANs is computationally expensive and requires careful balancing to avoid mode collapse.
Convolutional neural networks (CNN)-based denoising (85, 86)	CNNs are trained to identify and reduce noise in ECG signals automatically.	Efficient in removing structured noise while retaining ECG morphology; suitable for real-time applications.	Requires a large amount of labeled training data; computationally intensive.
Recurrent neural networks (RNN)-based denoising (85, 87)	Utilizes RNNs, particularly LSTMs, to handle time-series ECG data and remove temporal noise.	Effective in handling temporal dependencies and preserving important features such as R-peaks.	Training is computationally expensive and requires careful hyperparameter tuning to avoid overfitting.

#### 4.1.1 Classical filtering techniques (63–65)

Low-pass, high-pass, bandpass, and band-stop filters are the classical filtering techniques in ECG signal pre-processing. Bandpass filtering, which combines high-pass and low-pass filters, effectively removes baseline wander and high-frequency noise, preserving the heart’s signal within a specific frequency range. Notch filtering targets particular frequencies, such as 50/60 Hz powerline interference, to eliminate sinusoidal noise without affecting other aspects of the ECG signal. These methods are straightforward and widely used but can distort the ECG signal, especially in complex cases.

#### 4.1.2 Transform-based techniques (66–68)

Transform-based methods such as wavelet transform and empirical mode decomposition (EMD) offer advanced noise reduction by analyzing the ECG signal in different frequency components or intrinsic mode functions. Wavelet Transform is particularly useful for isolating noise from baseline wander and powerline interference by decomposing the signal into various frequency bands. EMD, on the other hand, adapts to non-linear and non-stationary signals by breaking the ECG signal into intrinsic mode functions, which can be selectively processed to reduce noise. These methods, while powerful, can be computationally intensive and may require careful parameter tuning.

#### 4.1.3 Statistical and adaptive techniques (69–71)

Statistical methods such as PCA and adaptive filtering focus on removing noise through statistical analysis and adaptive adjustments. PCA reduces dimensionality and noise by extracting significant components from multi-channel ECG data. Adaptive filtering, which uses reference noise signals to adjust filter coefficients dynamically, effectively addresses varying noise conditions, including powerline interference and motion artifacts (MArs). These techniques effectively reduce noise but may be limited in rapidly changing noise characteristics.

#### 4.1.4 Deep learning-based technique (85–87)

Recent advancements in machine learning have introduced sophisticated methods such as deep learning-based denoising, CNN denoising, and RNN denoising. These approaches leverage deep learning models to learn and remove complex noise patterns while preserving essential features of the ECG signal. For example, through adversarial training, GAN-based denoising techniques are used to separate noise from the ECG signal, enhancing adaptability to various noise types. CNN-based denoising uses convolutional layers to filter structured noise effectively. At the same time, RNN-based techniques, particularly those employing LSTM networks, manage temporal noise by capturing dependencies over time. Although these modern methods offer superior performance and adaptability, they require substantial computational resources and extensive training data to achieve optimal results.

#### 4.1.5 Advanced techniques (80–82)

Emerging methods such as sparse representation and transfer learning-based denoising provide innovative solutions for ECG signal pre-processing. Sparse representation filters out noise by representing the signal as a sparse linear combination of basis functions, which helps preserve signal morphology. Transfer learning-based denoising, utilizing pre-trained models, reduces the need for extensive data collection and training, making it efficient for small datasets. In addition, blind source separation (BSS) methods, such as independent component analysis (ICA), separate the ECG signal from noise sources by exploiting statistical independence, offering effective noise reduction in multi-lead ECGs. These advanced techniques continue to push the boundaries of ECG signal processing, addressing challenges that traditional methods may struggle with.

In ECG signal processing, significant noise artefacts pose a considerable challenge to the accurate analysis and interpretation of ECG signals. These extraneous disturbances can stem from various sources, including muscular activity, electromagnetic interference, electrode impedance, and baseline drift. Consequently, faithful extraction of relevant physiological information from noisy ECG recordings becomes critical, demanding innovative signal processing techniques (29). ECG recordings are generally mixed with different noises during acquisition from cardiac patients. Pre-processing techniques are crucial for the design of better classification systems. Identifying different fiducial points in ECG signals, such as P-onset and off-set, Q-onset and off-set, and T-onset and off-set, are difficult in noisy environments (91). Hence, the filtering of ECG signals without losing important information is a challenging task. The primary noise sources in ECG signal acquisition include MAs, caused by muscle contractions and tension, leading to irregular, high-frequency oscillations that distort the cardiac signal (7); electromagnetic interference (EMI) from electronic devices and power lines, causing voltage fluctuations and noise spikes (7); BW, gradual baseline shifts due to respiration or movement, obscuring low-amplitude cardiac components (92); PLI, visible as sinusoidal noise from electrical systems (9); MArs, high-frequency noise resulting from patient movements such as coughing or shifting positions (7); and electrode contact noise (ECN), resulting from poor electrode–skin contact, leading to signal distortion, especially during motion (2).

In addition, minor noise sources such as drift and offset, lead misplacement, sweat, moisture, and external interference also affect ECG morphology. Several researchers have developed different digital signal processing techniques to remove noise from the ECG signal (32, 38, 90, 93–96). In (93), an empirical mode decomposition was developed to effectively eliminate noise by using significant intrinsic mode functions of the ECG signal. A deep score-based diffusion model for ECG BW and noise removal and a multishot averaging strategy were developed to improve signal reconstructions in (96). A denoising autoencoder (DAE) was designed to remove the BW and PLI from the ECG signal in (95). A four-stage adaptive noise canceller was designed to remove the noise artefacts from the ECG signal in (94). In the dual stage, a different approach, namely singular value decomposition (SVD), was developed to improve the signal-to-noise ratio (SNR) of the ECG signal (38). Authors have proposed a sequential Monte Carlo algorithm combining the Wavelet transform to handle MA noise in the ECG signals (21). Other authors have proposed an R-peak detection and denoising algorithm using Shannon-energy and Hilbert transform (97). In the literature, several techniques have been developed to remove the noise from the ECG signals, even though there are still many challenges in real-time ECG acquisition. Some of the challenges are (i) patient movement during data acquisition can introduce noise and distortion to the ECG signal, making it difficult to extract the underlying cardiac information accurately; (ii) distinguishing between various types of noise and genuine cardiac signals is a critical step in effective noise removal as developing accurate algorithms for artifact identification is essential for preserving diagnostic integrity; and (iii) noise removal techniques developed in controlled laboratory settings may not always translate effectively to diverse clinical environments. Adapting and validating these techniques for real-world conditions are a challenge.

The pre-processing phase plays a pivotal role in ensuring that the ECG signal fed to machine learning or deep learning models is free from artifacts and retains physiologically relevant information. Raw ECG signals are typically contaminated by several types of noise, including baseline wander, PLI, MArs, and muscle (electromyogram) noise. Baseline wander, often caused by respiration or electrode movement, leads to low-frequency drift that can distort wave boundaries. Powerline interference introduces sinusoidal noise at 50 or 60 Hz, while MArs and muscle activity generate high-frequency components overlapping with the QRS complex, thereby degrading the diagnostic quality of the signal (38, 93). To mitigate these artifacts, various denoising approaches have been adopted. The Butterworth band-pass filter, commonly configured to between 0.5 and 40 Hz, effectively removes baseline drift and high-frequency disturbances while preserving critical cardiac information. Adaptive filters, such as the least mean square (LMS) (98) and recursive least squares (RLS) (48) algorithms, dynamically adjust their parameters to cancel correlated noise, especially powerline components and electrode movement artifacts (90). In addition, wavelet-based filtering has gained widespread use because it offers multi-resolution analysis. The discrete wavelet transform (DWT) (62) decomposes the ECG into different frequency bands, allowing selective thresholding to suppress noise without distorting QRS morphology (4). Normalization techniques, such as Z-score normalization and min–max scaling, are also applied to standardize ECG amplitude across subjects and devices, ensuring consistent model convergence. Across the reviewed literature, studies that employed multi-stage denoising pipelines—typically combining wavelet filtering with adaptive or Butterworth filtering—reported significant improvements in R-peak detection and classification accuracy. Overall, wavelet–adaptive hybrid pipelines consistently yielded superior signal quality and classification performance compared with single-stage filtering approaches (25, 57).

### 4.2 Feature extraction methods

Feature extraction techniques help to identify the different patterns and characteristics of the ECG signal, which can then be used to diagnose specific cardiac conditions. For example, feature extraction techniques can be used to identify abnormalities in the various segments of the acquired ECG signal, which can be indicative of different cardiac disorders. Therefore, accurate feature extraction and classification techniques are crucial for effective diagnosis and treatment. Section 4.2 reviews the existing techniques for feature extraction and classification of ECG signals in the literature.

#### 4.2.1 Handcrafted feature extraction

The performance of the classifier is dependent upon the extracted features. Feature extraction techniques are crucial in ECG signal processing and analysis, as they help identify important signal characteristics, such as amplitude, frequency, duration, and shape. Handcrafted feature extraction techniques are mainly classified into the following three types: time-domain (33), frequency-domain (33), and time-frequency domain (11) methods. These methods have shown promising results in identifying essential features of ECG signals that are useful for diagnosing different ECG beats.

#### 4.2.2 Time-domain features

Time-domain features refer to the signal characteristics based on the time each observation is measured. In other words, these features describe how the signal changes over time. Time-domain features include a signal’s mean, variance, and standard deviation and measures of the signal’s shape, such as its skewness and kurtosis. Some standard time domain features utilized in ECG analysis are RR interval, P-wave duration, QRS duration, QT interval, heart rate variability (HRV), PR interval, and ST segment. Clinicians use these time-domain features to diagnose and monitor a range of heart conditions, including arrhythmias, heart failure, and myocardial infarction. They can also be used in machine learning algorithms to develop automated classification models for detecting different types of ECG beats (4, 35, 91, 99–104). Time-domain features alone do not allow the model to better interpret the ECG signal and they provide limited information about the underlying physiological processes, as these features do not capture the complex patterns and dynamics of the ECG signal. These are sensitive to noise and artifacts, which can affect the classification’s performance. In addition, these are vulnerable to variations in signal morphology, such as changes in heart rate, respiration, and electrode placement. This can affect the accuracy and reliability of classification results. Several limitations need to be considered when developing a classification system. Combining time-domain features with other features or techniques is often necessary to improve classification performance (31).

#### 4.2.3 Frequency-domain features

Frequency-domain features are a group of traits or characteristics that characterize a signal’s frequency content. In signal processing, it is expected to work with signal representations in both the time and frequency domains. In contrast to time-domain features, which characterize the signal over a given time interval, frequency-domain features characterize the signal’s spectral characteristics throughout a range of frequencies. Some of the critical frequency-domain features include spectral power density (105), spectral entropy (106), spectral bandwidth (107), spectral flatness (108), and spectral skewness (109). Frequency-domain features can be extracted using various signal processing techniques such as the Fourier transform (FT) (105, 110), wavelet transforms (36), and spectrogram analysis (9). FT is a widely used technique for analyzing the frequency-domain characteristics of signals. In ECG analysis, the FT (110) can identify the frequency components in the ECG signal characteristic waves, such as the QRS complex, T wave, and P wave. The extracted features can be used for various applications, such as arrhythmia detection, heart rate variability analysis, and heart disease diagnosis. Frequency-domain features alone are not effective in classification due to the following reasons: some important features of the signal, such as the shape and duration of the QRS complex, may not be fully captured in the frequency domain; in some cases, different features of the ECG signal may have similar frequency components. This can make it difficult to distinguish between these features based on frequency-domain analysis alone. Frequency-domain analysis assumes that the signal is stationary over time, meaning its statistical properties do not change. However, ECG signals are often non-stationary, with features that change over time. In such cases, time-frequency analysis techniques may be more appropriate for ECG signal interpretation (62).

#### 4.2.4 Time-frequency domain features

Sections 4.2.2 and 4.2.3 individually covered the characteristics of the time and frequency domains for ECG beat classification systems. Classifying ECG beats accurately requires information from both the time and frequency domains. These features help capture the temporal and spectral characteristics of the ECG signal, which are crucial for distinguishing between different heartbeats. In ECG beat classification, time-domain features, such as the RR interval, QRS duration, and QT interval, are commonly used to extract information about the duration and amplitude of various segments of the ECG signal. However, these features do not provide information about the signal’s spectral content, which can be important when identifying specific types of heartbeats. In contrast, frequency-domain features provide information about the frequency content of the ECG signal. For example, the power spectrum of the ECG signal can be used to identify different frequency bands that correspond to specific physiological phenomena, such as the QRS complex, T wave, and P wave. In addition to time-domain and frequency-domain features, time-frequency features, such as wavelet transforms and spectrograms, are commonly used in ECG beat classification. These features provide a more comprehensive representation of the ECG signal, capturing both the temporal and spectral characteristics. The wavelet transform (111) is another technique that can be used to analyze the frequency domain characteristics of ECG signals. It provides a more localized frequency analysis than the Fourier transform and is useful in identifying transient features in the signal. DWT (29) is a signal processing technique that decomposes a signal into different frequency sub-bands. It is useful for identifying different frequency components in the ECG signal. These techniques can be used alone or in combination to extract frequency domain features from ECG signals. A number of transformation techniques can be utilized, i.e., dual-tree complex wavelet transform (DTCWT) (11), and Stockwell transform (ST) (29), to extract the time-frequency-based features from the pre-processed data. The short-time Fourier transform (STFT) uses a window function to analyze the signal in short-time intervals, which can lead to spectral leakage and reduced resolution. The resolution of the STFT is limited by the window size and the sampling rate, making it difficult to simultaneously analyze signals with high temporal and high-frequency content. Interpreting STFT results can be challenging, especially when analyzing complex signals with overlapping frequency content. The DTCWT requires careful selection of wavelet filters, which can be challenging and subjective. The DTCWT also requires significant computational power to process and analyze signals, especially for high-resolution applications or long signals. Finally, while the DTCWT offers improved shift-invariance compared to other wavelet transforms, it is not completely shift-invariant. This can cause issues in specific applications where shift invariance is critical. The ST provides high time-frequency resolution, allowing for a more accurate analysis of non-stationary signals. In addition, the S-transform is shift-invariant, meaning that it is not affected by signal translations or shifts in time, and it produces a two-dimensional (2D) representation of the signal in time-frequency space, which is easy to interpret and analyze. The ST can handle non-uniformly sampled data, making it suitable for applications where data are not uniformly sampled. Therefore, the ST is better compared to the STFT (31) and WT (11) for ECG beat classification (112).

#### 4.2.5 Deep learning-based feature set

Handcrafted feature extraction is a traditional method of extracting relevant features from raw ECG signals for ECG beat classification. The handcrafted feature engineering process is often time-consuming and requires significant effort and resources, which can hinder the development of large-scale systems (20). Another limitation is the potential for human bias. The features are designed by human experts, who may have inherent biases and subjective judgements. Finally, handcrafted feature extraction may not be suitable for complex ECG signals. The features are designed based on prior knowledge and assumptions about the data, which may not always hold in practice (18). This can result in features that are not representative of the underlying data distribution, leading to poor generalization and performance. Hence, automatic feature extraction based on deep-learning techniques from the ECG database has been introduced for ECG beat classification. Deep learning-based feature extraction has become an increasingly popular alternative to handcrafted feature extraction in recent years (113). Unlike handcrafted features, deep learning-based features are automatically learned from raw data, eliminating the need for human expertise and domain knowledge. Deep learning-based feature extraction involves training a neural network to learn a hierarchy of features from raw data. The first layers of the network learn simple, low-level features, such as edges and corners, while deeper layers learn more complex and abstract features. One of the main advantages of deep learning-based feature extraction is its ability to learn features tailored to the specific task. This contrasts with handcrafted features, which are designed based on prior knowledge and assumptions about the data (114). Once a deep neural network (DNN) has been trained on a large dataset, the learned features can be reused for other tasks or applied to new datasets. This can significantly reduce the time and resources required for feature engineering and model development.

Deep learning methods can be classified into several categories based on their architecture, learning mechanisms, and applications. Deep learning methods are generally categorized into three types, namely, discriminative, representative, and generative models (99). Discriminative deep learning methods are a class of deep learning algorithms designed to learn a mapping between inputs and outputs directly. Unlike generative models that learn the underlying probability distribution of the data, discriminative models learn to discriminate between different classes of data based on their features. Some popular discriminative deep learning methods include CNNs for classification, RNNs (25), and DNNs (9). These methods have achieved state-of-the-art performance in ECG beat classification. Discriminative deep learning methods typically involve many parameters learned through backpropagation. Backpropagation involves computing the gradient of a loss function for the model parameters and using it to update the parameters to minimize the loss. One of the advantages of discriminative deep learning methods is their ability to learn complex decision boundaries between classes, which can lead to high accuracy on classification tasks (26). However, they are often data-hungry and require large amounts of labeled training data to perform well.

Representative deep learning methods are essential for advancing deep learning because they are the foundation for developing new and innovative deep learning models. By understanding the underlying principles of these methods and the techniques used to optimize them, researchers can build upon them to create even more powerful and effective deep learning algorithms. Some examples of representative deep learning methods include GANs (115), autoencoders (AEs) (116), and deep belief networks (DBNs) (117). GANs are generative models that generate new data samples from a given input. They consist of two neural networks, a generator and a discriminator, trained in a minimax game (115). GANs have been successfully applied to tasks such as image generation, data augmentation, and anomaly detection. AEs are unsupervised deep learning models that are used for feature learning and dimensionality reduction (116). They consist of an encoder and a decoder network that learn to compress and reconstruct the input data. Autoencoders have been successfully applied to tasks such as image denoising, anomaly detection, and data compression. DBNs are deep generative models with multiple restricted Boltzmann machines (RBM) layers (117).

Generative deep learning models are a class of artificial neural networks (ANN) designed to generate new, synthetic data similar to data from a training set (115). These models can learn complex patterns and structures from the training data and then use that knowledge to generate new examples similar to the original data. Several generative deep learning models include variational autoencoders (VAEs) (118), GANs (115), and autoregressive models (AMs) (119). Discriminative models are designed to learn the boundary between different classes of data, while representative models aim to learn the underlying structure of the data. In contrast, generative models learn to generate new data similar to the training data. Overall, each of these three types of deep learning models has its strengths and weaknesses, and the choice of model depends on the specific task at hand and the nature of the dataset being used.

Feature extraction converts pre-processed ECG signals into compact and discriminative representations that can effectively describe cardiac morphology and rhythm. The extracted features generally fall into five categories, namely, time-domain, morphological, frequency-domain, time-frequency, and non-linear descriptors, each capturing different aspects of the ECG waveform (4). Time-domain features quantify temporal variations between successive heartbeats. Parameters such as the R–R interval, HRV indices [standard deviation of normal-to-normal intervals (SDNN), root mean square of successive differences (RMSSD), and percentage of successive normal-to-normal (NN) intervals that differ by more than 50 milliseconds (pNN50)], and mean or variance of beat-to-beat intervals reflect autonomic regulation and rhythm irregularities. These measures are computationally efficient and remain the foundation for arrhythmia detection in wearable and real-time systems (23). Morphological features describe the geometric and amplitude characteristics of individual ECG waves. Metrics including QRS width, P–R and Q–T intervals, R-peak amplitude, and area under the QRS complex capture structural deformation associated with ventricular and supraventricular ectopic activity. Derivative-based slopes and amplitude ratios between successive waves further enhance discrimination among beat classes (1).

Frequency-domain features, derived using the fast Fourier transform (FFT) (110) or power spectral density (PSD) (120), provide information on periodic energy distribution within specific frequency bands (0–40 Hz). However, because ECG signals are non-stationary, time-frequency representations such as the DWT (62), STFT (23), or wavelet packet transform (WPT) (36) are preferred. These methods capture transient spectral changes and localize abnormalities more accurately than pure spectral analysis. Finally, non-linear descriptors such as sample entropy, approximate entropy, and fractal dimension quantify signal complexity and chaotic behavior, while PCA (58) and ICA (121) are employed to reduce dimensionality and highlight salient features. Across the reviewed studies, hybrid feature sets combining wavelet coefficients with entropy-based complexity measures consistently achieved over 97% accuracy on the MIT-BIH Arrhythmia Database, underscoring the advantage of multi-domain representation for robust ECG beat classification.

### 4.3 Classification methods

In the ECG beat classification system, a classifier automatically classifies different heartbeats based on their ECG waveform features. Various machine and deep learning techniques have been reported for identifying different types of heartbeats (18–20, 30, 112, 114, 122–129). These classifiers utilized extracted features from the ECG signal to distinguish between different types of heartbeats. The classifier’s performance depends on the quality of the ECG signal, the feature extraction quality, and the choice of the classification algorithm. There are three different types of classification algorithms, namely, (i) unsupervised, (ii) semi-supervised, and (iii) supervised.

#### 4.3.1 Unsupervised

Machine learning can take the form of unsupervised learning, in which the algorithm can learn patterns and relationships in data without being explicitly supervised. In unsupervised learning, the algorithm receives a set of input data but no labels to indicate the desired results. The algorithm is trained to recognize patterns and connections in the data and cluster items with similar characteristics (130). The k-means clustering, hierarchical clustering, and PCA methods are all examples of popular unsupervised learning techniques (130). A machine learning clustering algorithm groups related data elements. Clustering partitions a dataset so that data points in the same cluster are more similar than those in other clusters. Clustering methods such as k-means, hierarchical, and density-based spatial are popular. Using the mean of data points inside each cluster, k-means clustering divides data into K clusters, iteratively assigning data points to the nearest cluster centroid and recalculating centroids until convergence. An algorithm in hierarchical clustering arranges data points into a tree-like structure of clusters, with each node representing a cluster. Each data point is initially treated as its own cluster by the algorithm, which then iteratively merges the two closest clusters together until all data points belong to the same cluster. The algorithm in density-based clustering organizes data into clusters according to their density, with points closer together having a greater density than those further away. To make a dataset more manageable, dimensionality reduction is another common unsupervised learning method. This can aid in making the data simpler and, therefore, easier to analyze. The standard methods include PCA and *t*-distributed stochastic neighbor embedding (t-SNE). PCA is an algorithm that projects data into a lower-dimensional space after determining the directions in the data with the most variance. This results in fewer features that can be used to represent the data. The t-SNE algorithm can reduce the dimensions in a dataset without losing the information about the relationships between the data points. This can be helpful when visualizing data at a higher level in a lower dimension. In (35), three independent, unsupervised techniques, namely, linear discriminant analysis (LDA), PCA, and ICA, were utilized to classify ECG beats, as per the AAMI standard. K-means clustering was used for the classification of premature ventricular contraction (PVC), normal (N), left bundle branch block (LBBB), paced beats (P), and right bundle branch block (RBBB) ECG beats in (131).

#### 4.3.2 Semi-supervised

Semi-supervised learning is a machine learning paradigm that falls between supervised and unsupervised learning. In the context of ECG beat classification, semi-supervised learning can be used to enhance the performance of the classification model by leveraging a large amount of unlabeled data (132). Several methods can be used for semi-supervised learning in ECG beat classification. One approach is to use a combination of unsupervised and supervised learning methods. In this approach, an unsupervised learning algorithm is used to cluster the unlabeled data, and then a supervised learning algorithm is used to classify the labeled data using the clusters as features. This can improve the accuracy of the classification model, as the unsupervised learning algorithm can identify underlying patterns in the data that may not be apparent to the supervised learning algorithm. Another approach is to use a generative model, such as a GAN, to generate synthetic labeled data from the unlabeled data. The synthetic data can be used to augment the labeled data and improve the accuracy of the classification model. In addition to these approaches, there are also active learning methods to select the most informative unlabeled samples for labeling (132). This can be particularly useful in scenarios where labeling the entire unlabeled dataset is not feasible due to time or resource constraints. Overall, semi-supervised learning is promising for ECG beat classification since it can use enormous amounts of unlabeled data to enhance the classification model’s accuracy (132). However, it is essential to thoroughly assess the model’s efficacy and ensure it can withstand shifts in the input data. To distinguish between SVEBs (also known as S beats) and VEBs (also known as V beats), Zahi et al. (37) proposed a semi-supervised iterative label update method. Semi-supervised strategies for the categorization of paroxysmal atrial fibrillation (PAF) using CNNs and LSTMs are reported in (133).

#### 4.3.3 Supervised

Unsupervised and semi-supervised learning are not typically the best approaches for ECG beat classification because they rely on clustering or dimensionality reduction techniques, which may not capture the complex and diverse patterns in ECG signals (134). ECG signals can contain various beat types and subtypes, each with distinct characteristics. Unsupervised and semi-supervised learning techniques may have difficulty accurately identifying and separating these different beat classes. Unsupervised and semi-supervised learning techniques are often used when labeled data are limited or expensive. The effectiveness of semi-supervised learning depends heavily on the quality of the unlabeled data. If the unlabeled data contain a lot of noise or irrelevant information, it can decrease the model’s performance. In addition, semi-supervised learning requires a subset of the data to be labeled, which can be time-consuming and expensive. Determining which data to label can also be challenging to maximize the model’s performance. ECG beat classification is a critical task that requires a high degree of accuracy (134). Supervised learning approaches are better suited to this task as they can be trained to optimize for accuracy and can leverage a larger number of labeled data points. However, in the case of ECG beat classification, a substantial amount of labeled data is available, making supervised learning approaches a more appropriate choice. The algorithm is trained in supervised learning using a labeled dataset containing examples of ECG signals and their corresponding beat types. The algorithm aims to learn a function that maps the input ECG signal to the correct beat type (134).

Some of the supervised machine learning classifiers are ANNs (122), SVMs (135), Hidden Markov models (127) and self-organizing maps (SOMs) (136). Hidden Markov models are used to detect cardiac arrhythmias, as reported in (4). DTCWT is utilized to extract the morphological features and merge them with the temporal features. Five different types of ECG arrhythmias have been categorized using a multi-layer back propagation (MLP-BP) neural network (137). Regarding neurons in the deep layers, MLP-BP is extremely sensitive. Underfit occurs in MLP when the number of neurons in the hidden layer is low. Too many neurons in the hidden layer may cause the fitting curve to oscillate erratically due to overfitting. The network model will stop functioning if the weights are high. Although DTCWT shows merit as a feature extraction strategy, the method’s final classification performance suffers from the limitations of the MLP-BP algorithm (9). To improve SVM’s generalization capability in the identification of various ECG beats, particle swarm optimization (PSO) is employed (138). In (29), five distinct types of ECG beats were classified using an algorithm based on bacteria foraging optimization (BFA) and SVM. Using SVMs, Pawel et al. (122) proposed an ensemble classifier for categorising arrhythmias. In this case, a genetic algorithm was used to optimize the characteristics acquired by Weich and the discrete Fourier transform (DFT). In (123), various classifiers, including naive Bayes, linear and quadratic discriminating functions, and J48 classifiers based on majority voting, were used to categorize five distinct heartbeats according to the AAMI standard. To extract information from an ECG signal, (139) used a DWT in conjunction with a novel one-dimensional hexadecimal local pattern (1D-HLP) approach and then used a single nearest-neighborhood (1NN) classifier to categorize 17 different types of arrhythmias. The genetic ensemble of classifiers optimized by sets (GECS) was used to categorize 17 myocardial dysfunctions in (124). We estimate power spectral density features to improve the quality of the ECG signal. Feature extraction, the process of choosing and extracting valuable features from the ECG signal for use in machine learning methods, is performed manually. However, the classifier’s precision may suffer if inappropriate features are used. Knowing which features are the most important when performing a classification task can be difficult. There have been several proposals for classifying cardiac arrhythmias, but many of the efforts that have been reported to date have at least one of the following limitations: (i) accuracy was only good for a few carefully chosen ECG recordings; (ii) feature extraction methods were overly complicated; (iii) classifier performance was suboptimal; (iv) fewer output classes; and (v) beat loss when the ECG signal was filtered for noise.

The use of deep learning algorithms for ECG beat classification has increased in recent years. CNNs (18), RNNs (25), DBNs (117), AEs (116), and attention-based models are only some of the deep-learning methods that can be applied to the problem of ECG beat classification. Automatic feature extraction is the main advantage of deep learning models. In recent years, deep-learning models have modified their structure in ECG beat classification to improve accuracy. During the initial stages of ECG beat classification, the prevailing models relied on handcrafted features, such as QRS duration, heart rate, and T-wave amplitude. These models exhibited a restricted level of accuracy, prompting researchers to initiate investigations into deep learning models. CNNs have achieved notable success in classifying ECG beats due to their ability to directly extract relevant attributes from the raw ECG signal, minimizing the requirement for manually extracted features. In an initial study by (127), a one-dimensional CNN (1D CNN) was introduced. This CNN is capable of accurately identifying ECG beats without the need for manually extracted features. However, this research employed an FFT to preprocess the ECG beats. In (18), a 1D-CNN was introduced to process raw ECG signals without pre-processing. In (140), a parallel configuration of CNN is described as an efficient method for classifying ECG beats. Following the emergence of CNNs and RNNs, these models were developed to process data sequences effectively. RNNs can effectively capture the sequential dependencies and extended patterns found in ECG data.

A deep neural LSTM including spectral features for ECG data classification has been suggested by Grzegorz et al. (113). In (26), different ECG beats were detected simultaneously using a combination of a CNN and LSTM. In (141), four types of ECG beats were classified using a dense convolutional network (DenseNet) and bi-directional long short-term memory (Bi-LSTM) architecture that combines the wavelet transform. Furthermore, the research on ECG beat classification has also included other combinations, such as CNN-LSTM (27), LSTM-CNN (27), CNN-BiLSTM (142), and Bi-LSTM (25). DL algorithms have the ability to acquire sophisticated attributes and comprehend intricate patterns in ECG signals. ECG beat classification is especially crucial when significant deviations in beat morphology exist. Therefore, DL-based methods (142–148) are outperforming previously published techniques such as template matching, rule-based, and ML-based methods (12–17, 22–24). Most of the methods outlined in the current literature have limitations, such as feature extraction that requires human intervention, issues with class imbalance, requiring a large amount of training data, and the need for powerful GPUs.

A proficient hybrid deep learning architecture is required for efficient automatic feature extraction and ECG beat classification. 1D-CNNs are efficient but require more depth and parameters to achieve higher accuracy. ResNet was designed to address this problem, but overfitting may arise when the model’s structure is very intricate, requiring high-performance hardware and extended training duration. Furthermore, ResNet is unsuccessful in capturing the long-term dependencies in a sequence. In the majority of the studies in the literature, researchers convert one-dimensional ECG beats into grey-scale images (39) or spectrograms (112) to improve performance. However, this approach can be computationally expensive, particularly when working with images rather than signals. Thus, a bi-directional gated recurrent unit (Bi-GRU) is implemented to capture the long-term dependencies in an ECG signal. Bi-GRU has the ability to process the input sequence in both forward and backward directions. This enables the model to effectively capture contextual information from past and future inputs, making it highly valuable for tasks that involve analyzing the relationships within the input ECG beat segment.

Bi-GRU is more efficient in training and converges faster than ResNet, particularly when working with smaller datasets (28). This is due to its reduced parameter count. Although Bi-GRU may not be able to extract complex features from input data like CNNs and ResNet, it still has its strengths. Using a CNN, ResNet, or Bi-GRU alone fails to improve ECG beat classification performance. To address these problems, the utilization of dual-stream or multi-stream (40) networks can be beneficial for achieving precise classification of ECG beats. Utilizing dual or multi-stream deep learning techniques to combine information from multiple sources or modalities can greatly enhance the performance of deep learning models (28). In dual-stream deep learning, the model receives data from two sources, each representing a distinct input or feature. In one stage in the model’s design, these streams are usually combined to produce a forecast. Multi-stream deep learning is quite similar but uses three or more data streams. This approach may be particularly beneficial when dealing with complex data that can be broken down into numerous modalities. It is now common practice to employ dual or multi-stream deep learning approaches to combine data from many sources to enhance models’ predictive abilities (28).

An optimal fit in a machine learning or deep learning model is considered good because it strikes a balance between underfitting and overfitting, allowing the model to generalize well to unseen data (144). Unlike underfitting, where the model is too simple to capture important patterns, or overfitting, where it memorizes noise from the training data, an optimal fit ensures that the model learns meaningful relationships without excessive complexity (163). This results in better generalization, reduced bias and variance, improved accuracy on both training and validation datasets, and enhanced robustness across different data distributions. By maintaining this balance, an optimally fitted model provides reliable and stable predictions, making it suitable for real-world applications where consistency and adaptability are essential.

Traditional machine learning classifiers continue to play an essential role in ECG beat classification owing to their interpretability and computational efficiency. Among these, SVMs and ensemble models, such as random forest and gradient boosting, remain dominant, achieving accuracies of 98%–99% when combined with optimized DWT–PCA features. Simpler methods, such as kNN, decision tree, and naïve Bayes, provide lightweight alternatives for embedded or real-time systems. Addressing class imbalance through SMOTE or cost-sensitive learning further enhances reliability. Overall, ensemble-based and kernel-optimized SVM frameworks deliver a strong balance between accuracy, speed, and interpretability, confirming that well-engineered ML systems remain competitive with deep-learning models in ECG beat classification.

## 5 ECG beat classification using advanced machine learning

We thoroughly analyzed several articles that use machine learning, deep learning, and explainable AI (XAI) techniques to classify ECG beats. To address this, we have included a summary note on AI-based methods for ECG diagnosis in Section 5.

### 5.1 Machine learning approaches

An overview of previous studies on the classification of ECG beats through the application of machine learning techniques is presented in Table 6. The table reviews various ECG beat classification techniques using machine learning models, highlighting their applications, databases, and performance metrics. Techniques such as radial basis function neural networks (RBFNNs), SVMs, and ensemble classifiers have been applied in datasets such as MITDB, PTBDB, and INCART, achieving accuracy rates from 84.60% to 99.99%. Each approach has specific advantages, such as resilience to noise and improved specificity, but also faces limitations, including increased system complexity or computational demands. Notably, classifiers such as SVM and RF consistently showed high performance, making them suitable for real-world applications; however, challenges in feature selection and model generalization were critical issues in different studies. After extracting the features from the ECG beats, artificial intelligence methods from machine learning can be used to build models from these data to classify arrhythmia heartbeats (43, 43–46). SVMs (126), ANNs (164), KNNs (24), and LDAs (165) are some of the most popular machine learning methods used for ECG beat classification. Despite noise and outliers in the ECG data, SVM continues to produce effective results because it finds the ideal hyperplane that maximizes the margin between distinct classes. Basic artificial neural network structures with fewer layers and parameters could improve interpretability, making it easier for clinicians to comprehend and have confidence in the model’s decisions when classifying ECG beats. The KNN algorithm is beneficial due to its simplicity and non-parametric nature, which makes it suited for small-to-medium-sized datasets and circumstances with extremely non-linear decision boundaries (24). However, when the classes in the ECG data are well-separated, LDA emerges as a linear classifier that strives to maximize the between-class variation while minimizing the within-class variance (165). Moreover, it is important to consider the dataset’s size, the task’s difficulty level, and the available processing resources before selecting an approach. Each algorithm has both advantages and disadvantages.

**Table 6:** Review of the ECG beat classification techniques that use ML models.

Study	Application	Database	ML approach	Performance (in %)	Advantages	Disadvantages
(149)	Arrhythmia classification		RBFNN	Sen 94.54	Optimized classification performance with minimal network size.	A minimal number of morphological features are extracted from ECG beat.
(150)	ECG beat classification		LDA and MLPNN	Acc 84.60 Acc 89.00	Non-linear classification capabilities were improved.	Generalization capability of the system needs to be improved.
(151)	ECG beat classification		SVM	Acc 97.20	Resilient to noise and variability.	Limited generalization capability.
(152)	ECG beat classification		LR	Acc 98.43 Spe 97.75	A high average specificity of 97.75% with minimal false positives.	Implementing and fine-tuning Reservoir Computing algorithms are complex.
(153)	AF classification	MITDB	SVM	Acc 90.27 F1 84.00	Suited for real-world applications.	AF rhythm detection has a lower F1 score.
(154)	Abnormalities detection		Ensemble classifier	Acc 90.25	Improves the classification system’s performance.	May require enormous computational resources.
(155)	ECG classification		CNN with SVM	Acc 97.10 Sen 96.50	Improved performance with CNN and SVM.	Complex model requiring extensive tuning.
(156)	Cardiac arrhythmia detection		EEMD with KNN	Acc 93.40 Sen 95.40 F1 96.30	Supports efficient automatic diagnosis in clinical settings.	Advanced signal processing increases computational demands.
(157)	ECG beat classification		Decision tree Gradient boosting	Acc 98.62 Acc 99.13	Good accuracy for early heart disease detection.	Noise and artifacts may affect performance.
(35)	Five types of ECG beat classes		PCA, LDA, ICA, DWT	Acc 99.97	DWT offers energy compaction; choice of dimensionality reduction depends on data characteristics.	Not tested with 10-fold validation; lacks generalization as tested only on MITDB.
(9)	Five beat classification		RF classifier	Acc 98.50	Higher accuracy with 10-fold validation.	DTCWT for feature extraction is hard to implement on hardware.
(29)	ECG beat classification	INCART	SVM	Sen 91.70	Improved classification performance, and generalization capability of the model is verified.	System complexity increases with various connected components.
(158)	Arrhythmia classification	MITDB SVEB	KNN and SVM with PSO optimization	Acc 94.50 Acc 85.10	Maintains high accuracy for 15 heartbeat classes.	Higher computational cost due to PSO optimization.
(159)	Heart diseases	MITSA	RF GDB	Acc 97.98 Acc 96.95	Acceptable for large datasets.	Performance may drop with poor feature selection.
(24)	Myocardial infarction & heart failure	NSR, PHR, MIT-BIH sinus arrhythmia	KNN	Acc 98.40	Effective with small datasets.	Memory-intensive for large datasets.
(126)	ECG anomaly detection	Physionet long-term ECG	Gaussian kernel-based SVM classifier	Acc 99.99	Handles high-dimensional spaces effectively.	Computationally expensive with large datasets.
(160)	Myocardial infarction	PTBD	SVM	Acc 95.30 Sen 94.60 Spe 96.00	Consistently better results.	Computationally intensive model.
(161)	Myocardial infarction	PTB	SVM, KNN, and RF	Sen 92.60 Sen 92.30 Sen 91.43	Automated MI diagnosis.	Multiple-instance learning complicates classification.
(92)	ECG arrhythmia detection	SVEB	SVM with Kruskal-Wallis Feature selection	Acc 98.06 MCC 91.51	Robust on imbalanced datasets.	Preprocessing may limit generalizability.
(162)	Apnea detection	Private	CHMM	Sen 93.98 Spe 95.38	Works well with real-time data.	Limited training data reduces performance.

MLPNN, Multilayer Perceptron Neural Network; EEMD, Ensemble Empirical Mode Decomposition; CHMM, Coupled Hidden Markov Model; IraNet, Inter- and Intra-patient Representation Aggregation Network.

ECG beat classification using machine learning classification algorithms increases cardiac health assessment efficiency, accuracy, and automation. Initially, these methods offered rapid and automated ECG data analysis, saving healthcare workers time and effort. This may improve heart abnormality diagnosis and treatment outcomes and lead to lower healthcare expenditures. In addition, machine learning classifiers can efficiently process significant ECG data, making scalable and cost-effective analysis possible in clinical settings. Furthermore, these methods can understand intricate patterns and correlations within ECG readings, which enables the detection of minor anomalies that may not be evident to traditional human observers.

However, machine learning algorithms for ECG beat classification have been associated with notable challenges and limitations. These models depend on the quality and quantity of training data, which may be noisy, artifact-filled, and variable among populations. In addition, the sophisticated decision-making processes of machine learning models in ECG analysis may be difficult for clinicians to understand. This misinterpretability may limit the clinical acceptance of machine learning-based diagnostic technologies.

### 5.2 Deep learning approach-based ECG beat classification

A comprehensive literature review on ECG beat classification using deep learning models is presented in Table 7. The review of deep learning models for ECG beat classification revealed various approaches, such as CNNs, LSTMs, and hybrid models, applied to datasets such as MITDB, INCART, and SVDB. CNNs show robust performance with accuracies up to 99.90%, while LSTM-based methods excel in capturing temporal features, improving sensitivity and specificity. The advantages of these models include the automation of feature extraction and their potential for real-time applications. However, challenges remain, such as computational intensity, model complexity, and limited generalization across diverse datasets. Ensuring robustness in noisy, real-world conditions and addressing resource-intensive training remains critical for their wider clinical adoption. Deep learning has become an essential tool in ECG beat classification, with enormous significance in medical diagnostics and healthcare (18, 26). A key advantage of deep learning over more conventional machine learning approaches for ECG beat classification is its ability to automatically derive hierarchical features from raw ECG data, eliminating the need for features to be manually extracted (18, 26). In contrast, deep learning models, specifically CNNs and RNNs, are exceptionally proficient at automatically extracting complex features from raw ECG data, which allows for the accurate classification of a wide range of cardiac disorders and arrhythmias (18, 19).

**Table 7:** Review of the studies on ECG beat classification using deep learning models.

Study	Application	Database	DL approach	Performance (in %)	Advantages	Disadvantages
(127)	ECG beat classification		CNN	Acc 97.40 Sen 60.31	DL replaces time-consuming, error-prone manual feature extraction.	Training CNNs for each patient can be computationally intensive, especially with more patients.
(136)	ECG heartbeat classification		CNN (nine-layered)	Acc 94.30 Acc 84.07 (Noisy ECG)	Accurately classifies heartbeats in noise-free ECGs, indicating ability to detect abnormal rhythms.	Training dataset quality significantly impacts performance. A biased dataset can hinder model generalization.
(137)	ECG arrhythmia detection		DCNN (seven-layered)	Spe 99.83 Acc 99.68	More efficient and accurate than conventional methods.	Limited generalization to other datasets or real-life clinical scenarios.
(135)	ECG arrhythmia classification		1D-CNN 2D-CNN	Acc 90.93 Acc 99.00	2D-CNN model classified five arrhythmias accurately.	High computational resources and time needed for training, especially for 2D-CNN.
(166)	ECG heartbeat classification		LSTM with AE	Sen 98.63 Spe 99.66	LSTM-AE learns features without manual extraction.	LSTM networks are computationally expensive to train and deploy.
(129)	ECG beat classification	MITDB	LSTM-based RNN	Acc 99.10 F1 95.00	Patient-specific analysis achieved by training on 5 min segments.	Complex implementation hinders adoption, especially with limited resources.
(167)	ECG arrhythmia classification		Multiscale convolution and FCBA (frequency convolutional block attention)	Acc 95.60 Sen 93.17	CNN with new multiscale blocks and attention modules classify arrhythmia with high sensitivity and accuracy. Effectively resolves data imbalance using oversampling and noise augmentation.	Complex model architecture and preprocessing methods may need huge computational resources. The method’s performance may change with increasingly diversified datasets.
(39)	Normal atrial fibrillation		Encoded deep CNN with input ECG beats as image	Acc 99.52 F1 Score 95.64	The morphological variations can be captured more effectively using images compared to one-dimensional signals.	Complex models with high computational power (GPU-based systems) are required to handle the large input image data.
(40)	Beat classification as per AAMI standard		BiLSTM and random forest with PCA	Acc 98.30	Ability to extract more in-depth features from the ECG signal.	As it is an ensemble learning model, it requires more training and testing time.
(28)	Beat classification as per AAMI standard		Multi-stream Bi-GRU network with random forest	Acc 99.93	Deep features are extracted from the input, which are very helpful to accurately assess the type of beat.	The reliance on multiple deep-learning models can lead to high computational costs. Further research is needed to evaluate scalability for real-time applications.
(141)	ECG arrhythmia classification		DenseNet with Bi-LSTM	Acc ∼99.44	BiLSTM is integrated to enhance the model’s capacity for extracting local features and capturing temporal features of ECG signals.	Bi-LSTM requires more computational power and needs to process input twice due to the parallel deep learning architecture.
(27)	ECG arrhythmia classification (six types)		CNN and LSTM	Acc 99.32	The proposed model exhibits strong generalization capabilities and could serve as a valuable tool for clinicians in diagnosing arrhythmia.	Requires QRS detection, introducing additional computational overhead. Imbalanced dataset challenges, particularly with limited AFL class instances.
(10)	Beat classification as per AAMI standard		An explainable deep transfer learning approach	Sen 99.00	Explainable AI enhances the accuracy and reliability of heartbeat classification by making DL models more transparent and understandable for physicians.	Deployment feasibility for real-time monitoring must consider computational resources, integration with clinical workflows, and regulatory standards.
(168)	ECG arrhythmia classification	MITDB AFDB	Customized CNN	Acc 97.31 Sen 96.50 F1 98.30	Classifies numerous arrhythmias accurately utilizing short ECG segments, making them appropriate for continuous monitoring using wearable devices.	Real-world wearable data noise and artifacts may affect performance. Single-lead ECG may lower arrhythmia detection accuracy.
(143)	Detection of atrial fibrillation	AFDB	CNN and RNN combination	Acc 89.30	Extract high-level features from segments of RR intervals (RRIs) to classify them as either AF or NSR.	Analysis of the model’s performance on noisy ECG segments revealed a higher number of false positives, as anticipated.
(169)	ECG signal Classification	MITDB ICCAD	CLINet (Conv+LSTM+ Involution)	Acc 99.94 Acc 99.90	The architecture is lightweight, making it appropriate for deployment on wearable devices, and it achieves very high accuracy across multiple datasets.	A lack of comprehensive preprocessing may influence robustness in noisy real-world datasets.
(170)	ECG beat classification	MITDB INCART SVDB	DNN	Acc 91.30 Acc 92.40 Acc 90.61	Enhanced system robustness and generalizability.	Stacking autoencoders and DNNs is computationally expensive and resource-intensive.
(171)	Premature ventricular contraction	MITDB INCART	Hybrid BiLSTM	Acc 97.20 Sen 96.00	Achieves high performance in classifying premature ventricular contractions.	May be less effective in detecting rare arrhythmias or artifacts.
(172)	ECG beat classification	LUDB	VGG16-based CNN	Acc 99.90	Accurately classifies beats using time-frequency representation.	Impressive accuracy may indicate overfitting, especially on smaller datasets.
(173)	Abnormalities detection	MITDB Real-time	Hybrid DNN	Acc 99.28 Acc 99.12	High accuracy ensures reliable abnormality detection for clinical applications.	Heavily reliant on input data quality; noise and artifacts can reduce accuracy.
(170)	ECG beat classification	MITDB INCART SVDB	DNN	Acc 91.30 Acc 92.40 Acc 90.61	Enhanced system robustness and generalizability.	Stacking autoencoders and DNNs is computationally expensive and resource-intensive.
(174)	Heartbeat classification	SVEB	IraNet (residual attention with Bi-LSTM)	Acc 95.48 Sen 95.75	Competitive accuracy and sensitivity across multiple classes.	Complex model may require extensive computational resources.

DL algorithms can improve diagnostic ability by utilizing extensive datasets, allowing them to generalize across various cardiac diseases and adjust to differences in patient demographics and recording settings (18, 26). Over the past few years, CNNs have been utilized extensively in ECG diagnosis and attained remarkable performance. CNNs are superior to other methods for extracting spatial characteristics from input signals; this allows them to detect local patterns that may indicate different cardiac problems. In contrast, RNNs can understand the temporal correlations between subsequent ECG samples since they specialize in modeling sequential data (25–27). By utilizing the combined advantages of convolutional neural networks and recurrent neural networks, particularly in hybrid structures such as CNN-RNN, ECG classification algorithms can attain exceptional performance in terms of accuracy and robustness (26–28).

DL methods have various advantages over standard ECG beat classification algorithms, including automatically learning discriminative features from raw ECG data without manual feature extraction (18). With this automated feature extraction technique, DL models can better classify cardiac diseases by detecting intricate patterns and tiny variations in the data. Further, the temporal and sequential nature of ECG signals is well-suited to DL methods, especially RNNs and CNNs, enabling them to detect the long-range relationships and temporal patterns in heartbeats (25). Furthermore, DL models have also shown excellent generalization abilities, which means they can adapt to different patient groups and recording situations, which is very important for real-world clinical applications.

In the field of ECG beat classification, deep learning algorithms have some significant limitations and challenges. Overfitting is a significant challenge in ECG beat classification, particularly in deep learning-based models, where the model captures noise and irrelevant patterns from training data, leading to poor generalization to new, unseen data. One of the primary causes of overfitting in ECG classification is the use of complex models with high-dimensional feature spaces, especially when datasets are imbalanced. Studies have shown that utilizing all 12 ECG leads in classification models can introduce redundancy and unnecessary complexity, thereby reducing generalizability and increasing the risk of overfitting (176). In addition, the presence of linear dependencies within ECG data has been identified as a major issue that skews the learning process towards the majority class, leading to biased model predictions and lower accuracy in minority class detection (57). This issue is particularly evident in patient-specific ECG classification, where models tend to overfit to the training patients, failing to generalize effectively across different individuals (29). Moreover, deep learning models trained on high-dimensional spectro-temporal ECG features have been found to suffer from overfitting due to their inability to leverage beat-to-beat variations effectively (120).

To mitigate overfitting in ECG beat classification, researchers have proposed various strategies. Feature selection and dimensionality reduction techniques, such as selecting an optimal subset of ECG leads instead of using all 12 leads, have been found to enhance classification accuracy while maintaining interpretability (176). Sample reduction techniques, such as QR and SVD, have also been effective in eliminating redundant data and addressing class imbalance, thereby reducing bias and improving generalization (57). In addition, the use of ensemble methods, such as multiple classifier architectures and hybrid machine learning approaches, has shown promise in improving classification robustness and interpretability (60, 175). Regularization techniques, including dropout and L2 regularization, are widely used to constrain deep learning models and prevent overfitting. Cost-sensitive learning approaches, such as modifying the classification threshold based on class imbalance, have also been shown to significantly enhance ECG beat classification performance by reducing bias towards majority classes (98). Furthermore, data augmentation strategies, such as adding noise, applying transformations, or generating synthetic ECG beats using generative models, have been successful in increasing the diversity of training data and improving model generalization (120). Collectively, these approaches ensure the reliability and robustness of ECG beat classification models, making them more effective for real-world medical applications.

In addition to the overfitting, large annotated datasets are also necessary for effective deep neural network training, which is a significant challenge. Generating high-quality annotated ECG data, particularly for rare cardiac disorders, can be costly and time-consuming (9). Furthermore, DL models are computationally demanding and require enormous computational resources, particularly during the training and optimization phases. This may limit their scalability and practical implementation in contexts with limited resources, such as point-of-care settings. Despite these challenges, continuous research is endeavoring to overcome these constraints by creating interpretable DL architectures, data-efficient learning methods, and hardware optimizations (24). The ultimate goal is to maximize the benefits of DL for ECG beat classification and simultaneously reduce its potential drawbacks.

Deep learning has become the leading paradigm for ECG beat classification by automatically learning spatial-temporal features from raw signals. Among various models, CNN-LSTM hybrids and attention-enhanced networks consistently demonstrate superior performance, achieving accuracies of 98%–99% across multiple ECG databases. CNNs capture morphological patterns, while LSTM and GRU layers model temporal dependencies. Transformer-based models further improve interpretability and long-range context handling. Data augmentation with GANs and representation learning via autoencoders effectively address class imbalance and noise. In addition, transfer-learning and lightweight architectures, such as MobileNet, enable real-time deployment on wearable devices. Overall, hybrid and attention-driven frameworks represent the state of the art in robust, generalizable ECG beat classification systems.

### 5.3 Explainable AI for ECG beat classification

ECG beat classification using XAI leverages advanced machine learning techniques to enhance transparency in automated diagnostic systems. Traditional classification methods often function as “black boxes,” making it difficult for clinicians to interpret or trust decisions, especially in critical applications such as arrhythmia detection. Explainable AI addresses this limitation by employing methods such as saliency maps, feature importance visualization, and decision boundary analysis. These approaches allow for better understanding and verification of the features contributing to a model’s predictions. Techniques such as layer-wise relevance propagation (LRP) and gradient-weighted class activation mapping (Grad-CAM) have been applied to visualize critical regions of ECG signals, helping practitioners align model decisions with medical insights (177, 178). This not only improves diagnostic reliability but also aids in identifying potential errors in classification. Incorporating XAI into ECG beat classification aligns automated systems with real-world clinical requirements. Explainable methods enable the assessment of model predictions in handling class imbalances and morphological complexities in ECG datasets. Studies integrating XAI with neural networks, such as convolutional and recurrent architectures, have shown improved interpretability without compromising classification accuracy (58, 121). For instance, combining ICA with neural networks has demonstrated robust classification results with enhanced feature interpretability, achieving over 98% accuracy on benchmark datasets (98, 179). By bridging the gap between machine intelligence and clinician trust, explainable AI ensures that automated ECG diagnostic tools are not only effective but also transparent and clinically viable. A detailed comparison of the different features is summarized in Table 8.

**Table 8:** Comparison of XAI, traditional ML, and deep learning.

Feature	XAI	Traditional ML	Deep learning
Transparency	High transparency; models provide insights into decision-making processes.	Moderate transparency; some models (e.g., decision trees) are interpretable, others (e.g., SVM) are not.	Low transparency; often acts as a “black box.”
Accuracy	Slight trade-off in accuracy due to constraints of explainability in some cases.	Moderate accuracy depending on the model and dataset.	High accuracy, especially in complex tasks.
Interpretability	Clear reasoning behind predictions, improving trust and adoption.	Varies; interpretable in simple models (e.g., linear regression) but not in ensemble models.	Poor interpretability due to complexity.
Scalability	Dependent on the complexity of explainable methods; may require additional computation.	Good scalability; lightweight and efficient.	Highly scalable for large-scale data.
Application areas	Suitable for trust-sensitive fields like healthcare and finance.	General-purpose applications like classification and regression.	Ideal for unstructured data such as images, text, and audio.
User trust	Builds trust through interpretable results.	Moderate; depends on model interpretability.	Limited due to black-box nature.
Complexity of implementation	Higher due to integration of methods like SHAP, LIME, or Grad-CAM.	Moderate; simpler than XAI and DL.	High; requires expertise in network architecture and optimization.
Regulatory compliance	Aligned with regulations requiring transparency and accountability.	Limited compliance where explainability is required.	Poor compliance due to lack of interpretability.
Performance on unstructured data	Performs well but may lag behind DL in raw accuracy.	Moderate; pre-processing required for unstructured data.	Excels with unstructured data such as images and signals.

XAI has emerged as a crucial frontier in ECG beat classification research, bridging the gap between high-performing deep-learning models and clinical interpretability. While deep neural networks, such as CNNs, LSTMs, and transformers, have demonstrated outstanding diagnostic accuracy, their “black-box” nature continues to limit real-world clinical trust and regulatory acceptance. Among the 106 reviewed studies, approximately 14% (15 papers) explicitly incorporated XAI methods to interpret model predictions and visualize decision reasoning. Most of these studies applied saliency-based techniques, including Grad-CAM (180) and LRP (60), to highlight the waveform segments that contribute most strongly to the classification outcomes. Such visualization maps frequently revealed correspondence between algorithmic focus regions and clinically significant components of the ECG—particularly the QRS complex, P-wave, and ST–T segments—demonstrating the potential of XAI to validate model reasoning in physiological terms.

Beyond saliency methods, a smaller number of studies utilized attention mechanisms within hybrid CNN-LSTM architectures to provide interpretability by weighting critical temporal regions. Others employed the SHapley Additive exPlanations (SHAP) (181) and Local Interpretable Model-Agnostic Explanations (LIME) to quantify feature importance in models trained on handcrafted attributes. However, the overall adoption of such explainability frameworks remains limited. Approximately 86% of the analyzed deep-learning studies focused solely on predictive performance metrics—accuracy, precision, recall, or F1-score—without providing interpretive insight into model behavior or physiological relevance. This significant gap highlights that the ECG-AI community remains primarily performance-driven, with interpretability often treated as an auxiliary consideration rather than a core design principle. The limited integration of XAI tools underscores an urgent need for standardization in interpretability reporting. Future research should emphasize quantitative explainability benchmarks, such as region relevance overlap with annotated ECG segments, and clinician-in-the-loop validation to assess whether the model explanations align with expert reasoning. Integrating XAI at the design stage can enhance clinical transparency, regulatory compliance, and physician confidence, ultimately enabling safer and more explainable deployment of AI-driven cardiac diagnostic systems.

## 6 Limitations, solutions, and future directions

Though this review provides a comprehensive overview of ECG beat classification systems, it is important to acknowledge several limitations inherent in the current ECG beat classification approaches.

### 6.1 Minor challenges

- As the traditional methods use handcrafted features, they are unable to provide reliable and adequate information from similar ECG beats due to the action of morphological patterns, which may lead to misclassification (11, 43–46).
- Deep learning models involve many computations, particularly those with large architectures such as CNNs or RNNs. High-end GPUs offer more powerful processors and parallel processing capabilities, enabling faster and more efficient execution of these computations than CPUs (central processing units) (20, 25, 28, 114).
- Deep learning algorithms require extensive training datasets to train the models (9, 19).

*Solutions for minor challenges:* Implementing deep learning models, such as CNNs and RNNs, that automatically learn relevant features from raw ECG data can significantly improve classification accuracy (127). These models capture complex patterns and morphological variations without relying on handcrafted features. To optimize neural network architectures for computational efficiency, techniques such as model pruning and quantization can be adopted, or lightweight models such as MobileNet can be utilized (182). Leveraging cloud-based GPU resources or distributed computing helps meet computational demands without compromising performance (183). Employing data augmentation techniques to artificially expand the training dataset by introducing variations, such as scaling, rotation, or adding noise to existing ECG signals, enhances the model’s robustness (184). In addition, one could use transfer learning from models pre-trained on large datasets or explore semi-supervised learning methods that can effectively use unlabeled data (185).

### 6.2 Major challenges

- ECG signals exhibit significant intra- and inter-patient variability in beat morphology due to differences in heart anatomy, electrode placement, noise, artifacts, and physiological factors. This variability makes it challenging for deep learning models to generalize across different individuals and accurately classify ECG beats (18, 20, 28, 116).
- Most of the pre-processing techniques in the literature depend on the signal sampling frequency. If the sampling frequency of the input signal varies, it is very difficult to achieve better classification accuracy (113, 114).
- Deep learning models generally require a large amount of labeled data to achieve high accuracy. Obtaining a large and accurately annotated ECG dataset for training deep learning models can be challenging, as ECG data collection and annotation are time-consuming and require expertise (10, 25, 136).
- ECG beat classification models trained on one dataset may not generalize well to unseen data from different sources. Ensuring the robustness and generalizability of deep learning models is a challenge in ECG beat classification (9, 18, 19).
- The performance of the classification algorithms is significantly degraded in the presence of similar morphological patterns with minor variations from different classes (9, 19, 26, 27).

*Solutions for major challenges:* Collecting a diverse dataset that represents different patient populations is crucial for training models that generalize well (186). Techniques such as domain adaptation can adjust the model to new patient data, and personalization strategies can fine-tune the model based on individual patient characteristics (187). Standardizing the sampling frequency by resampling all ECG signals to a common rate prior to processing ensures consistency (188). Developing pre-processing methods that are robust to sampling frequency variations or designing models that handle inputs with different sampling rates using adaptive algorithms can mitigate issues related to varied sampling frequencies (189). The use of semi-supervised learning and active learning approaches reduces the amount of labeled data required (190). Collaborating with medical institutions to share and pool annotated datasets expands the available training data (191). Exploring synthetic data generation methods, such as the use of generative adversarial networks to create realistic ECG signals, provides additional training resources (192). Training models on combined datasets from multiple sources improves generalization capabilities (163). Implementing cross-validation strategies across different datasets and using domain generalization techniques ensure that models are robust to variations in data distribution from different sources (193). Enhancing the model’s ability to detect subtle differences by incorporating attention mechanisms and multi-scale feature extraction techniques improves discrimination between classes with similar morphological patterns (194). Using ensemble methods that combine multiple models further enhances classification performance (195), and fine-tuning the model using specialized loss functions, such as focal loss, emphasizes hard-to-classify examples (196).

### 6.3 Synthesis of the proven solutions and best practices

From the 106 selected articles, we identified consistently successful practices for ECG beat classification, particularly regarding data imbalance, generalization, model design, and evaluation (55). A key observation across numerous studies is that models trained and tested on the same patient cohort (intra-patient splits) tend to exhibit inflated performance, as they inadvertently learn subject-specific morphology (127). In contrast, inter-patient validation—where all beats from a given subject appear in only one data split—offers a more realistic assessment by ensuring generalization to unseen individuals. Therefore, inter-patient (or leave-one-subject-out) evaluation should be considered the primary reporting standard, with any intra-patient results clearly labeled as ablation or sensitivity analyses. The synthesis of reviewed literature highlights that rigorous data curation and proper dataset partitioning contribute significantly more to trustworthy and generalizable results than methodological novelty alone. These best practices address four major recurring challenges in ECG beat classification: data imbalance, architectural design, training and evaluation methodology, and reproducibility. Therefore, it is increasingly accepted that inter-patient evaluation should serve as the primary reporting protocol, with intra-patient results presented only as supplementary ablation tests. Equally important is the transparent documentation of any pre-processing, i.e., band-pass filtering (BPF) parameters, baseline-wander removal, R-peak detection methods, and beat segmentation window lengths, since small differences in these steps can significantly alter results (57). Explicitly reporting such settings is now recognized as best practice for reproducibility.

The most persistent problem identified across datasets such as MIT-BIH Arrhythmia (8), INCART (53), and MIT-BIH Supraventricular Arrhythmia (3) is the severe class imbalance between normal and abnormal beats. To mitigate this, studies have increasingly combined algorithmic and data-level solutions. Among the algorithmic solutions, loss-function re-weighting or dynamic minority-biased batch weighting consistently improved sensitivity to rare arrhythmias without harming majority class precision. These methods dynamically scale gradients according to class frequency, preventing the network from converging toward trivial majority predictions. Complementary strategies such as feature-fusion architectures, which integrate temporal, frequency-domain, and time-frequency representations, were also effective, as they expose the classifier to richer discriminative cues and mitigate bias towards dominant waveform shapes (197). A smaller but influential group of studies used DBA or F1-based early-stopping criteria instead of overall accuracy, thereby explicitly optimizing for balanced recognition across classes. Collectively, these practices demonstrate that metric-aware optimization is more faithful to clinical relevance than simple accuracy maximization. At the data level, the combination of ECG-specific augmentation and transfer learning emerged as another reliable route to improved minority-class recall. Augmentations such as controlled time-shifting, amplitude scaling, additive Gaussian noise, and mild temporal warping expand training diversity while preserving physiological plausibility. Overly aggressive distortions, such as heavy frequency modulation, were found to degrade performance by altering beat morphology. Consequently, augmentations that respect signal physiology are now favored. For smaller or domain-specific datasets, transfer learning—pre-training on a large balanced corpus and fine-tuning on the target data—consistently reduced overfitting and variance, especially for rare arrhythmias. Ensemble learning, though computationally heavier, repeatedly provided robustness against minority misclassifications by averaging the decisions of differently initialized or architected models (21). Together, these results establish a hierarchy of preferred imbalance-handling practices, namely, loss-level weighting, physiologically consistent augmentation, transfer learning, and lightweight ensembles, in order of practicality. Architectural trends in the literature show a clear evolution from manual feature engineering to end-to-end deep networks capable of automatically learning morphological and temporal patterns. Among these, 1D-CNNs remain the dominant backbone for local feature extraction because they effectively capture QRS-complex morphology and achieve high efficiency. When CNNs are extended with recurrent layers—especially LSTM or Bi-LSTM units—they gain temporal awareness, enabling them to exploit rhythm dependencies between consecutive beats. Hybrid CNN-LSTM architectures, sometimes referred to as hierarchical temporal models, consistently occupied the upper performance tier, with F-scores typically between 95% and 99% in benchmark datasets. These combinations proved particularly successful in distinguishing morphologically similar beats that differ primarily in timing rather than shape.

A further architectural enhancement repeatedly associated with improved interpretability and accuracy is the attention mechanism. Attention layers help the network emphasize diagnostically important regions of the ECG, such as the QRS onset and offset, while down-weighting redundant background segments. Across multiple studies, attention modules increased sensitivity to subtle morphological differences between ventricular and supraventricular ectopic beats, and they provided intuitive saliency maps that facilitate clinical interpretability. Where data and computational resources permitted, transformer-style multi-head attention further improved long-range context modeling, capturing dependencies across entire cardiac cycles rather than single beats. In parallel, modern convolutional refinements such as residual connections, squeeze-and-excitation blocks, and frequency-channel attention enhanced optimization stability and feature discrimination. These structural elements have therefore become standard components of competitive ECG classifiers. Beyond architecture, the review reveals consensus on several training and evaluation practices that determine whether good models generalize in clinically realistic conditions. Regularization through dropout, weight decay, and batch normalization is nearly universal among successful deep networks, preventing overfitting to small patient subsets. Early stopping based on validation F1-score or AUPRC proved more reliable than using loss reduction alone, because it guards against bias towards majority classes. Many articles also emphasize aligning model capacity with hardware constraints. For example, for wearable or edge deployment, compact 1D-CNNs with attention heads offer an attractive trade-off between interpretability, accuracy, and latency. Reporting inference time per beat or per signal window is increasingly viewed as essential, since real-time performance is critical for embedded medical systems.

### 6.4 Future directions

The proposed ECG beat classification techniques in the literature provide promising performance, but there is a need to develop such methods to overcome the limitations of the current techniques. In this section, we discuss some of the open possible extensions of the techniques proposed in this article.

- *Personalized, adaptive models for individualized ECG classification:* One significant future direction is the development of personalized and adaptive models that tailor the ECG classification to the individual patient’s data. Traditional models often struggle with variability in ECG signals across different patients due to factors such as age, physiology, and comorbid conditions. By incorporating patient-specific data into the training process, models can adjust their parameters to better reflect individual cardiac patterns. Transfer learning and online learning can fine-tune models using a patient’s historical ECG data, leading to more accurate and reliable diagnoses (198, 199).
- *Explainable and interpretable deep learning models:* Deep learning models must be both explainable and interpretable for widespread clinical acceptance. Clinicians must understand the reasoning behind a model’s prediction to trust and effectively use it in decision-making. Future research should focus on integrating explainable AI techniques, highlighting which parts of the ECG signal contribute the most to a classification decision. Methods such as attention mechanisms, saliency maps, and layer-wise relevance propagation can provide insight into the inner workings of the model (181, 200). By transparentizing the decision process, we can bridge the gap between complex algorithms and clinical practice, ensuring that these tools support rather than hinder healthcare professionals.
- *Efficient, real-time processing for wearable and mobile devices:* As wearable technology and mobile health applications become more prevalent, there is a growing need for algorithms that can process ECG data efficiently and in real-time. Future developments should aim to optimize models to run on devices with limited computational resources without sacrificing accuracy. This can be achieved through model compression techniques, such as quantization and pruning, and the design of lightweight architectures specifically tailored for edge computing (182, 201). Efficient algorithms enable continuous monitoring and prompt detection of cardiac anomalies, which is essential for timely medical responses and for improving patient care in everyday settings.
- *Unlabeled ECG data with advanced learning techniques:* A vast amount of ECG data remains unlabeled due to the time and expertise required for annotation. Unsupervised, semi-supervised, and self-supervised learning techniques offer ways to utilize this untapped resource. Future research should focus on developing models that can learn meaningful representations from unlabeled data, thereby reducing the dependence on large labeled datasets. Techniques such as autoencoders, contrastive learning, and generative adversarial networks can uncover underlying patterns in the data, which can then be fine-tuned with minimal labeled examples (202, 203). Leveraging unlabeled data not only enhances model performance but also accelerates the development of robust ECG classification systems.
- *Performance with federated learning:* Patient privacy is of paramount concern in healthcare, limiting data sharing across institutions. Federated learning presents a solution by allowing models to be trained on decentralized data without transferring sensitive information. Future directions involve implementing federated learning frameworks for ECG analysis, enabling collaboration between hospitals and research centers while maintaining patient confidentiality (180, 197). This approach can lead to the creation of more generalized models trained on diverse datasets, improving accuracy and reliability across different populations. Addressing challenges such as communication efficiency and model convergence in federated settings will be essential for practical deployment.
- *Multi-modal learning:* Combining ECG data with other physiological signals or medical information through multi-modal learning can significantly improve diagnostic precision. Future work should explore integrating data from sources such as blood pressure monitors, oxygen saturation sensors, and patient medical histories. By providing a more comprehensive view of a patient’s health status, models can make more informed predictions about cardiac events (204, 205). Multi-modal approaches can help identify complex conditions that may not be detectable through ECG analysis alone, leading to more holistic and effective patient care. Developing algorithms capable of processing and synthesizing information from multiple modalities will be a key area of focus.

## 7 Limitations of the review methodology

Despite the systematic and comprehensive approach adopted in this review paper, several methodological limitations must be acknowledged to ensure transparency and contextual accuracy in the interpretation of our findings. These limitations arise from the nature of systematic reviews themselves, the search and selection procedures, the heterogeneity of the included studies, and the diversity in experimental designs, databases, and performance metrics used in the studies on ECG beat classification. Recognizing these constraints provides a balanced understanding of the review outcomes and outlines potential directions for improving future systematic reviews in this field.

### 7.1 Scope and search limitations

The review process followed the Preferred Reporting Items for Systematic Reviews and Meta-Analyses (PRISMA) guidelines to ensure methodological rigor and reproducibility. Nevertheless, one inherent limitation stems from the restriction to peer-reviewed, English-language publications indexed in major databases such as PubMed, IEEE Xplore, ScienceDirect, and Springer. While this criterion ensured quality and accessibility, it potentially excluded significant research reported in non-English journals, regional repositories, theses, and conference proceedings not indexed in the selected databases. Excluding non-English studies may have introduced a language bias, especially given that ECG research is conducted globally, with active contributions from Asian, European, and South American institutions. Consequently, important innovations, particularly those published in national medical journals or non-indexed conference proceedings, may have been overlooked. This review paper provides a detailed analysis of machine learning and deep learning techniques for the classification of ECG beats, focusing on the advancements from 2014 to 2024, but it may have excluded earlier pioneering work that laid the groundwork for more advanced ECG analysis methods. A systematic approach was adopted to analyze the 106 studies, offering a comprehensive evaluation of methodologies and their applications in ECG classification. Another limitation relates to the deliberate exclusion of grey literature, including technical reports, dissertations, white papers, and non-peer-reviewed conference abstracts. While this approach enhanced the credibility of the included studies by ensuring they met peer-review standards, it may have resulted in the omission of innovative but unpublished work or early-stage algorithmic implementations. Grey literature often contains valuable methodological insights, comparative evaluations, or negative findings that are less likely to appear in journal publications due to publication bias.

A major methodological limitation of this review arises from the heterogeneity across the datasets used in the included studies. The three most frequently employed databases—MIT-BIH Arrhythmia (8), St. Petersburg INCART (53), and MIT-BIH Supraventricular Arrhythmia (3)—differ substantially in sampling frequency, signal resolution, lead configuration, and patient demographics. Consequently, performance metrics such as accuracy, sensitivity, and F1-score cannot be directly compared across studies, as the underlying data distributions vary considerably. For instance, the MIT-BIH Arrhythmia database contains 48 half-hour two-lead ECG recordings from 47 subjects sampled at 360 Hz, whereas the INCART dataset (53) includes 75 recordings at 257 Hz from 25 patients with different arrhythmia profiles. These discrepancies introduced variability in the reported results, even when identical algorithms are used. Furthermore, some studies utilized patient-specific evaluation protocols (intra-patient validation), while others employed inter-patient cross-validation strategies. The choice between these validation schemes has a profound effect on reported accuracy. Models evaluated using intra-patient splits typically yield inflated performance metrics since the test data shares morphological characteristics with the training data. Conversely, inter-patient validation better represents real-world generalization but usually yields lower accuracy. The lack of standardized evaluation protocols across the studies makes it difficult to provide an entirely uniform assessment of algorithmic performance.

## 8 Recommendations from the critical review

Based on our comprehensive systematic review of 106 studies on the state-of-the-art machine and deep learning techniques for ECG beat classification, we propose the following recommendations to advance the field and address existing limitations. Addressing the prevalent issue of data imbalance, techniques such as SMOTE, GANs, and transfer learning should be employed to enhance model performance on underrepresented arrhythmia classes. Robust pre-processing pipelines using modern denoising methods, such as deep learning-based filtering and wavelet transforms, are crucial for mitigating noise artifacts such as baseline wander and motion interference. Hybrid and ensemble models that combine handcrafted and deep learning-based features can leverage domain knowledge while improving classification robustness. The adoption of transformer architectures and attention mechanisms can better capture long-term dependencies in ECG signals, while patient-specific and adaptive models, utilizing transfer learning and federated learning, can address inter-patient variability. To enhance real-world applicability, optimizing models for deployment on wearable and Internet of Things (IoT) devices, with a focus on lightweight architectures and edge computing, is essential. Furthermore, standardizing evaluation metrics and benchmarking datasets, fostering interdisciplinary collaborations, integrating multimodal data sources, and emphasizing ethical considerations such as data privacy and XAI will collectively ensure the development of clinically relevant and scalable ECG classification systems. These recommendations aim to address current challenges in ECG beat classification and pave the way for innovative, clinically relevant, and scalable solutions. By focusing on these areas, researchers and practitioners can enhance the diagnostic accuracy and real-world applicability of automated ECG analysis systems.

## 9 Conclusions

This systematic review highlights growing trends in deep learning for ECG classification, with CNN and hybrid models showing consistently high performance across benchmarks. Despite advancements, clinical translation is limited by bias, data imbalance, and lack of interpretability. While the field has witnessed significant progress, particularly with the adoption of deep learning methods such as CNNs and RNNs, challenges remain. These include noise and artifacts in ECG signals, data imbalance, the need for extensive annotated datasets, computational resource constraints, and the lack of generalizability to unseen data. In addition, many traditional approaches rely on handcrafted features, which may not fully capture the complexity and variability of ECG signals. Deep learning models have demonstrated superior performance in ECG beat classification tasks, offering the ability to automatically learn features from raw data and effectively capture temporal and morphological patterns. However, the “black-box” nature of these models raises concerns about interpretability, which is critical for clinical applications. Addressing these limitations requires the development of explainable AI algorithms that provide transparent decision-making while maintaining the high accuracy of deep learning models. This review underscores the need for robust pre-processing pipelines, advanced data augmentation strategies, and the integration of multimodal data to improve classification performance. Furthermore, interdisciplinary collaboration between researchers, clinicians, and engineers is essential to ensure that the developed systems align with clinical needs. By addressing these challenges and embracing emerging techniques, such as transfer learning, explainable AI, and real-time processing on wearable devices, future research can pave the way for more accurate, interpretable, and scalable ECG beat classification systems that improve patient care and diagnostic efficiency.

## Data Availability

The original contributions presented in the study are included in the article/Supplementary Material, further inquiries can be directed to the corresponding author.

## Glossary

ACC: Accuracy
AE: Autoencoder
AF: Atrial fibrillation
AFDB: Atrial Fibrillation Database
AHA: American Heart Association
AI: Artificial intelligence
AM: Autoregressive model
ANOVA: Analysis of variance
API: Application programming interface
AUC: Area under the curve
AUPRC: Area under the Precision–Recall curve
AV: Atrioventricular
BPF: Band-pass filter
BN: Batch normalization
BP: Blood pressure
BSS: Blind source separation
BW: Baseline wander
BWF: Baseline wander filter
CINC: Computers in Cardiology Challenge
CI: Confidence interval
CNN: Convolutional neural network
CNN-LSTM: Convolutional neural network combined with long short-term memory
CPU: Central processing unit
CPSC: China Physiological Signal Challenge
CSV: Comma-separated values
CV: Cross-validation
CVDs: Cardiovascular diseases
CWT: Continuous wavelet transform
DBA: Differential beat accuracy
DBN: Deep belief network
DNN: Deep neural network
DL: Deep learning
DOI: Digital object identifier
DTCWT: Dual-tree complex wavelet transform
DWT: Discrete wavelet transform
ECG: Electrocardiogram
ECN: Electrode contact noise
EEG: Electroencephalogram
EMD: Empirical mode decomposition
EMG: Electromyogram
EWT: Empirical wavelet transform
FCN: Fully convolutional network
FFT: Fast Fourier transform
FN: False negative
FP: False positive
FPGA: Field programmable gate array
GAN: Generative adversarial network
GRU: Gated recurrent unit
GPU: Graphics processing unit
HMM: Hidden Markov model
HPF: High-pass filter
HR: Heart rate
HRV: Heart rate variability
ICA: Independent component analysis
IMF: Intrinsic mode function
INCART: St. Petersburg Institute of Cardiological Technics Database
IoMT: Internet of Medical Things
IoT: Internet of Things
JCR: Journal Citation Reports
kNN: K-nearest neighbor
LMS: Least mean squares
LOSO: Leave-one-subject-out validation
LPF: Low-pass filter
LR: Learning rate
LSTM: Long short-term memory
MA: Muscle artifacts
MAr: Motion artifact
MAE: Mean absolute error
MFCC: Mel-frequency cepstral coefficients
MIT-BIH: Massachusetts Institute of Technology–Beth Israel Hospital Database
ML: Machine learning
MLP-BP: Multilayer perceptron-backpropagation
MSE: Mean squared error
NSTEMI: Non-ST-segment elevation myocardial infarction
NPV: Negative predictive value
PAC: Premature atrial contraction
PCA: Principal component analysis
PLI: Powerline interference
PPG: Photoplethysmography
PPV: Positive predictive value
PRISMA: Preferred Reporting Items for Systematic Reviews and Meta-Analyses
PTB: Physikalisch-Technische Bundesanstalt Diagnostic ECG Database
PVC: Premature ventricular contraction
PWA: Pulse wave analysis
Q: Quartile (Journal Citation Reports Ranking, Q1–Q4)
QOL: Quality of life
QRS: Q-wave, R-wave, and S-wave complex
RMS: Root mean square
RMSE: Root mean square error
RMSSD: Root mean square of successive differences
RNN: Recurrent neural network
ROC: Receiver operating characteristic
RR: R–R interval (interval between consecutive R-peaks)
SA: Sinoatrial (node)
SAE: Stacked autoencoder
SB: Sinus bradycardia
SD: Standard deviation
SDG: Sustainable development goal
SDNN: Standard deviation of normal-to-normal intervals
SJR: Scimago Journal Rank
SJR/Q: Scimago Journal Rank / Journal Citation Reports Quartile (Quality Index)
SMOTE: Synthetic minority oversampling technique
SNR: Signal-to-noise ratio
SOM: Self-organizing map
SoC: System on chip
Softmax: Softmax activation function
SPE: Specificity
ST: Stockwell transform
STEMI: ST-segment elevation myocardial infarction
STFT: Short-time Fourier transform
SVEB: Supraventricular ectopic beat
SVT: Supraventricular tachycardia
SVM: Support vector machine
SWT: Stationary wavelet transform
TL: Transfer learning
TCN: Temporal convolutional network
TN: True negative
TP: True positive
TQWT: Tunable Q-factor wavelet transform
VAE: Variational autoencoder
VEB: Ventricular ectopic beat
VF: Ventricular fibrillation
VT: Ventricular tachycardia
WT: Wavelet transform
ZCR: Zero-crossing rate
